# Synthesis and Structural Determination of New Brassinosteroid 24-Nor-5α-Cholane Type Analogs

**DOI:** 10.3390/molecules24244612

**Published:** 2019-12-17

**Authors:** Jocelyn Oyarce, Vanessa Aitken, César González, Karoll Ferrer, Andrés F. Olea, Teodor Parella, Luis Espinoza Catalán

**Affiliations:** 1 Departamento de Química, Universidad Técnica Federico Santa María, Avenida España 1680, Valparaíso 224000, Chile; jocelyn.oyarce@sansano.usm.cl (J.O.); vanessa.aitken.13@sansano.usm.cl (V.A.); cesar.gonzalez@usm.cl (C.G.); Karoll.ferrer.14@sansano.usm.cl (K.F.); 2 Instituto de Ciencias Químicas Aplicadas, Facultad de Ingeniería, Universidad Autónoma de Chile, El Llano Subercaseaux 2801, Santiago 8900000, Chile; andres.olea@uautonoma.cl; 3 Teodor Parella, Servei de Ressonància Magnètica Nuclear, Universitat Autònoma de Barcelona, 08193 Bellaterra, Barcelona, Catalonia, Spain; teodor.parella@uab.cat

**Keywords:** synthesis, brassinosteroid, analogues, 24-nor-5α-cholane

## Abstract

Natural brassinosteroids possess a 22*R*, 23*R* configuration that appears essential for biological activity. It is, therefore, interesting to elucidate if the activity of brassinosteroids with a short side chain depends on the C22 configuration. Herein, we describe the synthesis of new brassinosteroids analogs with 24-norcholane type of side chain and *R* configuration at C22. The initial reaction is the dihydroxylation of a terminal olefin that leads to *S*/*R* epimers. Three different methods were tested in order to evaluate the obtained *S/R* ratio and the reaction yields. The results indicate that Upjohn dihydroxylation is the most selective reaction giving a 1.0:0.24 *S*/*R* ratio, whereas a Sharpless reaction leads to a mixture of 1.0:0.90 *S*/*R* with 95% yield. Using the latter mixture and following a previous reported method, benzoylated derivatives and both *S* and *R* brassinosteroids analogs were synthesized. All synthesized compounds were completely characterized by NMR spectroscopy, and HRMS of new compounds are also given. In conclusion, a synthetic route for preparation of new analogs of brassinosteroids of 24-norcholane type and *R* configuration at C22 were described. It is expected that this will help to elucidate if a configuration at C22 is a structural requirement for hormonal growth activity in plants.

## 1. Introduction

**Figure 1:**
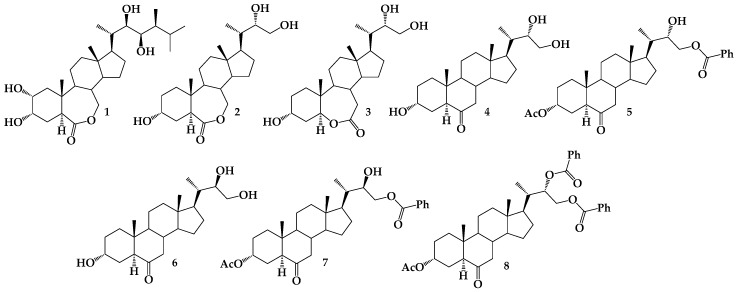
Structure of brassinolide (**1**) and analogs **2**–**8** of 24-nor-5α-cholan type.

Since the discovery of brassinolide (**1**) [1] (Figure 1) a large number of similar compounds have been identified in many plant species [2,3]. This family of polyhydroxylated sterol derivatives, named brassinosteroids (BRs), amounts to almost 80 different molecules which have been recently recognized as the sixth class of plant hormones [4,5]. They act as regulators of plant growth in very low amounts [1,2,3], participating in processes of cell expansion, division, and differentiation in young tissues of growing plants [6,7]. Additionally, they can protect plants from environmental stresses caused by salt, drought, and heavy metals [8,9].

The extremely low amount of BRs present in plants has prompted the chemical synthesis of BRs and 137 analogs have already been reported [4]. Structural variations of BRs arise from the type and position of functions in the A, B rings, fusion A/B ring, and alkyl side chain. One of the main synthetic difficulties is to get the alkyl side chain with the specific configuration found in natural BRs. According to the alkyl substitution in C24, BRs have been classified as C27, C28, and C29, and all natural bioactive BRs possess a vicinal 22*R*, 23*R* diol structural functionality that appears essential for high biological activity. It has been suggested that BRs may be derived from sterols with the same side chain, but most of the explored synthetic pathways require several steps with low yields. In recent decades, much effort has been dedicated to the synthesis of new BRs analogs in which common patterns of organic functions in the A/B rings and *cis–trans* fusion between them are kept intact, but with important structural changes in the side chain. For example, BRs analogs with shorter side chains [10,11,12,13], spirostanic and furostanic [14,15,16,17,18,19,20,21], aromatic and cyclic substituents [22,23,24,25,26,27], esters and carboxylic acids [24,28,29,30] have been reported. Despite these changes, these analogs have presented very important biological activity. Consequently, the structural requirements that these compounds should possess to elicit biological activity have changed and new SAR has been proposed [31,32]. Briefly, these results suggest that activity of BRs analogs depends on the oxygen atoms spatial distribution instead of the presence or absence of one specific functional group in the molecule [33,34]. These spatial orientations can be indicated as distances or angles between the oxygen atoms present in a brassinosteroid.

Following this line, we have previously reported the synthesis of BRs analogs with short side chain and structure (22*S*)-3α,22,23-trihydroxy-24-nor-5α-cholane (compounds **2**–**4**, Figure 1) and a benzoylated derivative **5** [35]. However, to evaluate the effect of a C22 configuration on biological activity it is necessary to obtain the 22*R* epimers of these analogs. Herein, we report on the synthesis of a new BRs analog and benzoylated derivatives with *R* configuration at C22. As the starting reaction is the dihydroxylation of a terminal olefin, synthesized from hyodeoxycholic acid, we have also evaluated the ratio of 22*S*/22*R* epimers obtained through three different dihydroxylation reactions. Yields of both possible configurations are compared for all proposed synthetic reactions.

## 2. Results and Discussion

**Scheme 1:**
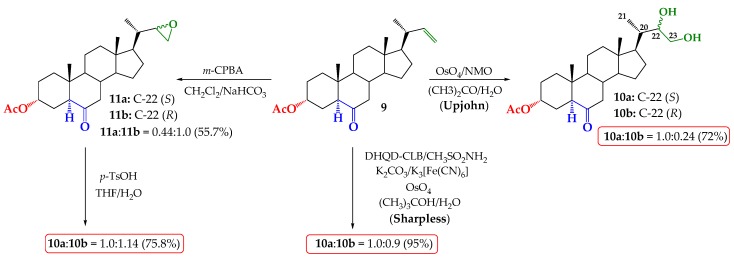
Dihydroxylation of olefin **9** to get a mixture of epimeric glycols **10a/10b** by using different synthetic pathways: 1) Direct Upjohn dihydroxylation; 2) Epoxidation and subsequent opening of epoxide ring in acid medium and; 3) Direct asymmetric Sharpless dihydroxylation.

The most direct synthetic route to BRs analogs with short side alkyl chains carrying a vicinal diol function is the dihydroxylation of terminal olefins. This transformation can be accomplished by either of three different methods, namely: Direct Upjohn dihydroxylation; Epoxidation and subsequent opening of epoxide ring in acid medium; and Sharpless asymmetric dihydroxylation (Scheme 1). Below are described the results obtained by the application of these synthetic paths to dihydroxylation of olefin **9**, which was previously synthesized from hyodeoxycholic acid [35]. All tested methods lead to epimeric mixtures of glycols 22*S*/22*R* (**10a**/**10b**) but in different ratios. In the following, a detailed description of reaction conditions and obtained yields are given for each method.

### 2.1. Direct Upjohn Dihydroxylation

Upjohn dihydroxylation is a well-known reaction that converts olefins to *cis* vicinal diol using *N*-methylmorpholine *N*-oxide (*N*MO) and osmium tetroxide. This reaction has been previously used to obtain glycols C22/C23 for 24-norcholane type steroids of similar structure. These studies have shown that the reaction product is a mixture of diastereomers with the 22*S* epimer as the major product [36,37,38,39]. Alkene **9** was hydroxylated by the Upjohn method and a mixture of epimeric glycols **10a**/**10b** was obtained with a 72% yield (Scheme 1).

**Figure 2:**
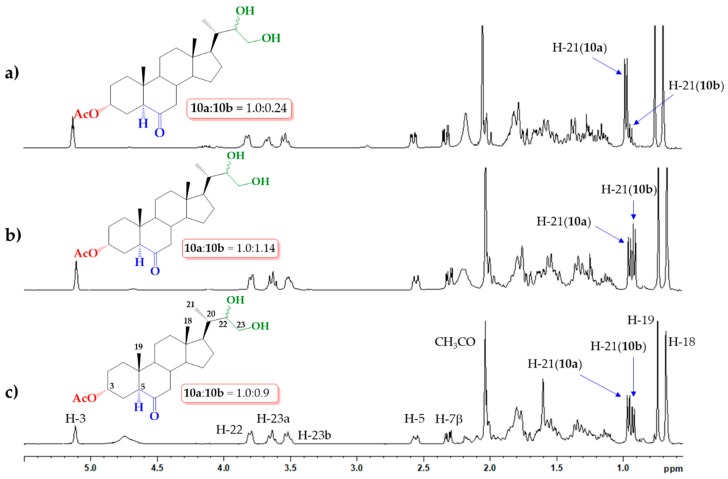
Spectra ^1^H-NMR of epimeric mixture of glycols **10a**:**10b** obtained by **a**) Upjohn method; **b**) Epoxidation and subsequent opening of epoxide ring in acid medium; **c**) Sharpless method.

The diastereomeric ratio of each glycol in the mixture can be established by integration of ^1^H-NMR signals assigned to the C21 methyl group, which appear at *δ*_H_ = 0.947 and 0.911 ppm in **10a** and **10b** epimers, respectively (Figure 2) [35].

Based on these NMR measurements the ratio of **10a**:**10b** obtained by this method was determined as 1.0:0.24.

### 2.2. Epoxidation and Subsequent Opening of Epoxide Ring in Acid Medium

It has been reported that the epoxidation reaction on steroidal nuclei of similar structure and carrying terminal double bonds C22/C23, mainly produces epoxide 22*R* [36,37,38]. Thus, epoxidation of alkene **9** with *m*-CPBA leads to a mixture of epimeric epoxides **11a**/**11b** with 55.7% yield (Scheme 1). The ratio in which these epoxides were obtained was **11a:11b** = 0.44:1.0, i.e., the amount of epoxide 22*R* is twofold larger than that of 22*S*. This proportion was calculated using the integrals of signals assigned to hydrogens H-22 (*δ* = 2.73 ppm), H-23a (*δ* = 2.59 ppm) of major epoxide (compound **11b**), and H-23a (*δ* = 2.64 ppm), H-23b (*δ* = 2.40 ppm) of the minor epoxide (compound **11a**) (see Figure S7, Supplementary Materials). For the determination of the majority epoxide, experimental and NMR spectroscopic data correlations were used [36,37,38]. Namely, it has been shown that comparative thin layer chromatography allows differentiating by polarity both epoxides formed in the epoxidation reaction of steroidal alkenes with 24-norchol-22-ene structures, and the results indicate that less polar epoxide corresponds to 22*S* configuration [36]. Our results show clearly that the more polar epoxide is preferentially obtained, i.e., epoxide **11b** with 22*R* configuration. Additionally, 1D and 2D NMR spectroscopy, particularly, 2D HSQC with edited phase (2D Edit HSQC), and 2D HMBC spectra, provided essential information to carry out assignments of H-21, H-22, H23a, H23b signals and all ^13^C signals present in the epoxide mixture **11a**/**11b.**

Subsequently, treatment of epoxides mixture **11a**/**11b** in acidic conditions using the *p*-TsOH/THF/H_2_O system (Scheme 1) produces **10a**/**10b** mixture with 75.8% yield [37,38]. Analysis of ^1^H-NMR spectrum indicates that the major component on this mixture is the less polar glycol 22*R* in the ratio **10a:10b** = 1.0:1.14 (Figure 2). This result indicates that the opening step favors the formation of diastereomeric ratio, changing the proportion of this epimer from 0.44 to 1.0.

### 2.3. Sharpless Asymmetric Dihydroxylation

Sharpless dihydroxylation reaction has not been used to obtain glycols C22/C23 in steroids with the shortest side chain of 24-nor-5α-cholane type. Thus, olefin **9** was dihydroxylated by using dihydroquinidine *p*-chlorobenzoate (DHQD-CLB) as a chiral ligand (Scheme 1), following the method described in reference [23]. The reaction product (95% yield) is a mixture of epimeric glycols **10a** and **10b** in the proportion 1.0:0.9, respectively (Figure 2).

In summary, none of the three tested methods led exclusively to one of the two epimeric glycols. The selectivity follows the order: Upjohn reaction produces mainly the glycol 22*S* in the ratio 1.0 to 0.24; a slightly reverse proportion is obtained by opening of epoxides, i.e., 1.0 to 1.14, whereas the Sharpless dihydroxylation reaction produced both glycols in similar amounts (1.0 to 0.9). The total yield of glycols mixture is also dependent of the dihydroxylation reaction: 95% for the Sharpless method, 72% for Upjohn, and 43% for expoxidation followed by opening of epoxide ring. Thus, a selection of one of these methods should depend on the expected outcome for hydroxylation reaction.

### 2.4. Synthesis of Benzoylated Derivatives

**Scheme 2:**
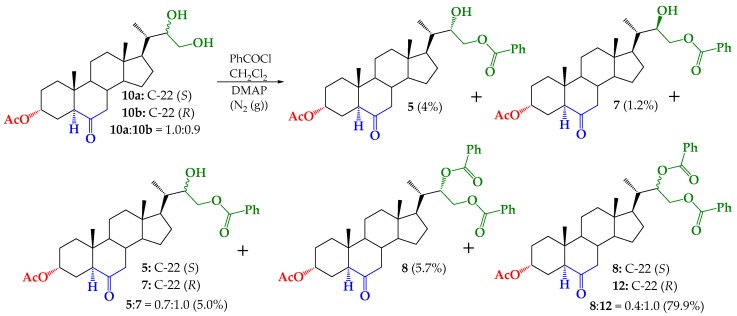
Benzoylated derivatives **5**, **7**, **8**, and **12** derivatives obtained by benzoylation reaction of glycols mixture 1**0a**/**10b**.

As the main goal of this research is to obtain derivatives with 22*R* configuration, we used the Sharpless dihydroxylation reaction to convert the olefin **9** in the mixture of epimeric glycols **10a/10b**. This reaction was selected because the mixture product is obtained with the highest total reaction yield (95%) and glycols **10a/10b** are found in similar proportion. The 22*S* benzoylated derivative (**5**) has been previously synthesized by reaction of pure compound **10a** with PhCOCl/DMAP [35]. Thus, to get compound **7** the glycols mixture **10a/10b** obtained by the Sharpless method was submitted to the same benzoylation reaction to obtain both epimers **5** and **7**. Interestingly, benzoylation of this mixture leads to a complex mixture of mono and dibenzoylated derivatives, i.e., compounds **5**, **7**, **8**, and **12** (Scheme 2). Compounds **5**, **7**, and **8** were purified by C.C. and recrystallized, giving yields of 4.0%, 1.2%, and 5.7%, respectively, whereas dibenzoylated derivative **12** could not be isolated in pure form. Additionally, mixtures of monobenzoylated epimers were also obtained, **5**/**7** with 5.0% yield (**5**:**7** = 0.7:1.0), and **8**/**12** with 79.9% yield (**8**:**12** = 0.4:1.0). The proportion of dibenzoylated epimers in both mixtures was determined based on the integration signals at *δ*_H_ = 1.22 and 1.16 ppm, assigned to the H-21 of methyl group for **8** and **12**, respectively.

#### Structural Determination of Derivatives **5**, **7**, and **8**

**Figure 3:**
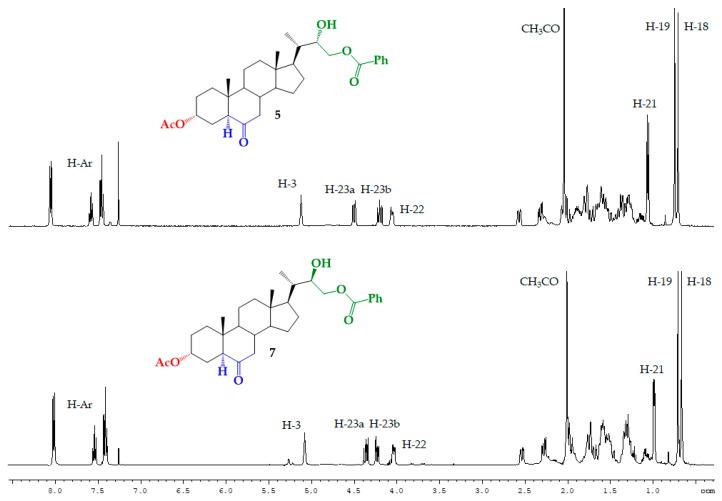
Comparison between ^1^H-NMR spectra of epimers **5** and **7**, and their main differences in chemical shifts for signals H-21, H-22, and H-23a–b.

The chemical structure of **5** has been previously determined by 1D and 2D NMR spectroscopy, whereas its absolute configuration 22*S* was established by using crystal X-ray diffraction techniques [35]. Thus, the structure and stereochemistry at C22 of compound **7** were established by a simple comparison of ^1^H and ^13^C-NMR spectra obtained for derivatives **5** and **7**. These comparisons considered chemical shifts (*δ*), coupling constants (*J*), and multiplicities of signals corresponding to H-22, H-23a, H-23b, and CH_3_-21. ^1^H-NMR spectra of both epimers are shown in Figure 3, and these signals are identified.

The main differences in these spectroscopic parameters are listed in Table 1, and the more significant variations in chemical shifts and *J* values correspond to hydrogen atoms H-23a and CH_3_-21. On the other hand, differences in chemical shifts were also observed in the ^13^C-NMR spectra, for signals of C21, C22, and C23. For the latter, the signals appear at *δ*_C_ = 66.39 and 68.84 ppm for **5** and **7**, respectively (Table 1). Based on this data the stereochemistry in epimer **7** was assigned as 22*R*.

**Figure 4:**
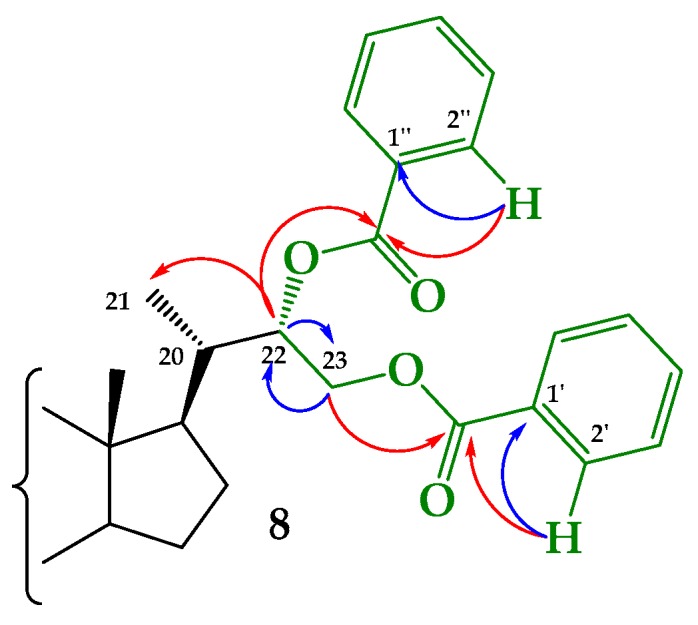
Main heteronuclear correlations 2D HMBC to ^2^*J*_CH_ (blue) and ^3^*J*_CH_ (red) observed for the dibenzoylated derivative **8**, which confirm the positions of both benzoate groups in C22 and C23 of the side chain.

The chemical structure of the dibenzoylated derivative **8** was established using ^1^H, ^13^C, 2D HSQC, and 2D HMBC NMR techniques. The presence of the dibenzoylated function was confirmed in the ^1^H-NMR spectrum, by signals observed at *δ*_H_ = 8.06 (2H, dd, *J* = 8.5 and 1.4 Hz); 7.97 (2H, dd, *J* = 8.5 and 1.4 Hz); 7.55–7.50 (2H, m); 7.44 (2H, dd, *J* = 7.9 and 7.9 Hz); 7.38 (2H, dd, *J* = 7.9 and 7.4 Hz), which are assigned to protons HAr-2″; HAr-2′, HAr-4″, and HAr-4′; HAr-3″ and HAr-5″; HAr-3′ and HAr-5′ in the aromatic group, respectively. In addition, in the ^13^C-NMR spectrum appear signals at 130.33; 133.06; 129.60; 128.35; 129.74; 133.00; 129.63, and 128.38 ppm, which were assigned to carbon C1′-Ar; C4′-Ar; C2′-Ar and C6′-Ar; C3′-Ar and C5′-Ar; C1″-Ar; C4″-Ar; C2″-Ar and C6″-Ar; C3″-Ar and C5″-Ar, respectively. These assignments were confirmed by 2D HSQC and HMBC spectra. The main structural information provided by the 2D HMBC spectrum was the assignment and position in the side chain of the two benzoate groups, which showed heteronuclear correlations at ^2^*J*_CH_ and ^3^*J*_CH_ as indicated in Figure 4.

To determine the stereochemistry at carbon C22 of compound **8**, a complete benzoylation reaction with PhCOCl/DMAP was carried out for the monobenzoylated derivative **5**. In this way, the dibenzoylated derivative was obtained with 95% yield. The spectroscopic data of ^1^H and ^13^C-NMR were totally consistent with those obtained for derivative **8** obtained by benzoylation of the glycol mixture **10a**/**10b**.

### 2.5. Synthesis of BRs Analogs

**Scheme 3:**
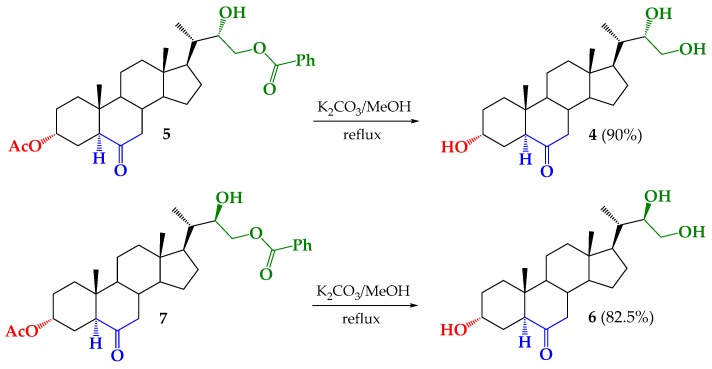
Synthesis of brassinosteroids (BRs) analogs **4** and **6** by saponification reaction of the monobenzoylated derivatives **5** and **7**, respectively.

Synthesis of BRs analogs **4** and **6** was carried out by saponification reaction of the monobenzoylated derivatives **5** and **7**, respectively, with 90% and 82.5% yield, respectively (Scheme 3).

**Figure 5:**
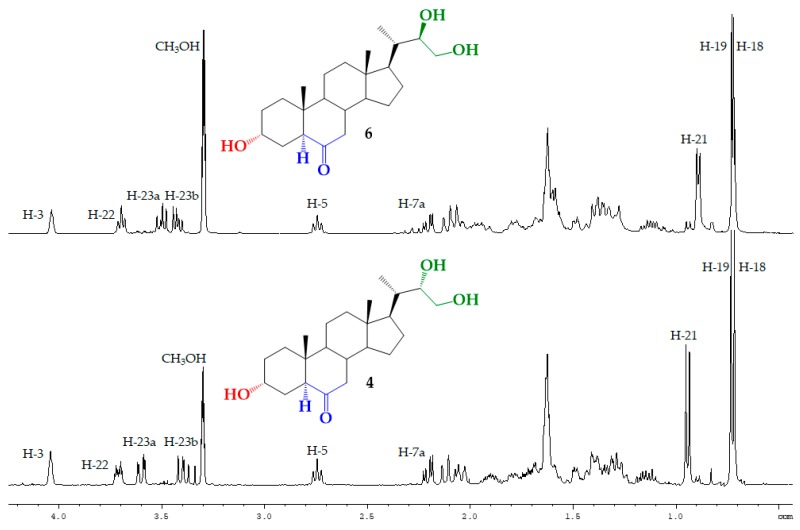
Comparison between ^1^H-NMR spectra of glycol epimers **4** and **6**, and their main differences in chemical shifts for signals H-21, H-22, and H-23a–b.

The spectroscopic data of ^1^H and ^13^C-NMR obtained for compound **4** were consistent with those previously reported [35,40]. The structural and stereochemical determination at C22 for triol **6** was established by comparison of NMR data between epimers **4** and **6**. These comparisons considered chemical shifts (*δ*), coupling constants (*J*), and multiplicities of signals corresponding to H-21, H-22, H-23a, and H-23b. ^1^H-NMR spectra of both epimers are shown in Figure 5, where these signals are identified.

The main differences observed among the indicated hydrogens are listed in Table 2. It is seen that hydrogen atoms H-21, H-23a, and H-23b experience variations in chemical shifts and *J* values, whereas H-22 shows also changes in multiplicity. On the other hand, in ^13^C-NMR spectra differences in chemical shifts for signals assigned to C21, C22, and C23, but mainly in carbon C21 and C23, are observed (Table 2). From this data the stereochemistry in C22 can be assigned as *R* for the epimer **6**.

## 3. Materials and Methods

### 3.1. General Experimental Methods

All reagents were purchased from commercial suppliers and used without further purification. Melting points were measured on a SMP3 apparatus (Stuart-Scientific, now Merck KGaA, Darmstadt, Germany) and are uncorrected. ^1^H, ^13^C, ^13^C DEPT-135, gs 2D HSQC, and gs 2D HMBC NMR spectra were recorded in CDCl_3_ or MeOD solutions, and are referenced to the residual peaks of CHCl_3_ at *δ* = 7.26 ppm and *δ* = 77.00 ppm for ^1^H and ^13^C, respectively, and CD_3_OD at *δ* = 3.30 ppm and *δ* = 49.00 ppm for ^1^H and ^13^C, respectively, on an Avance 400 Digital NMR spectrometer (Bruker, Rheinstetten, Germany) operating at 400.1 MHz for ^1^H and 100.6 MHz for ^13^C, and Avance 600 Digital NMR spectrometer (Bruker, Rheinstetten, Germany) operating at 600.13 MHz for ^1^H and 150.90 for ^13^C. Chemical shifts are reported in ppm and coupling constants (*J*) are given in Hz, multiplicities are reported as follows: singlet (s), doublet (d), doublet of doublets (dd), doublet of triplets (dt), triplet (t), quartet (q), multiplet (m), broad singlet (bs). IR spectra were recorded as KBr disks in a FT-IR 6700 spectrometer (Nicolet, Thermo Scientific, San Jose, CA, USA) and frequencies are reported in cm^−1^. High-resolution mass spectra (HRMS-ESI) were recorded in an Exactive Plus mass spectrometer (Thermo Scientific, Waltham, MA, USA). The analysis for the reaction products was performed with the following relevant parameters: heater temperature, 100 °C; sheath gas flow, 5 (arbitrary unit); sweep gas flow rate, 0 (arbitrary unit); and spray voltage, 3.0 kV at negative or positive mode. The accurate mass measurements were performed at a resolving power: 140,000 FWHM at range *m/z* 300–700. For analytical TLC, silica gel 60 in 0.25 mm layer was used and TLC spots were detected by heating after spraying with 25% H_2_SO_4_ in H_2_O. Chromatographic separations were carried out by conventional column on silica gel 60 (230–400 mesh) using EtOAc-hexane gradients of increasing polarity. All organic extracts were dried over anhydrous magnesium sulfate and evaporated under reduced pressure, below 40 °C.

### 3.2. Synthesis

#### 3.2.1. Upjohn Dihydroxylation of Alkene 9. Obtaining (22*S*)-22,23-dihydroxy-6-oxo-24-nor-5α-cholan-3α-yl acetate (**10a**) and (22*R*)-22,23-dihydroxy-6-oxo-24-nor-5α-cholan-3α-yl acetate (**10b**)

To a solution of alkene **9** (2.50 g, 6.47 mmol) in acetone (150 mL) *N*MO (0.45 g, 3.84 mmol) was added. After mixture homogenization by magnetic stirring, OsO_4_ (210 mmol) was added dropwise with stirring and reacted for 36 h at room temperature. The end of the reaction was verified by TLC. Then the solvent was removed (up to 25 mL approximate volume) and water (25 mL) and Na_2_S_2_O_3_^.^5H_2_O (25 mL saturated solution) were added. The organic layer was extracted with EtOAc (2 × 30 mL), washed with water (2 × 50 mL), dried over MgSO_4_, and filtered. The solvent was evaporated under reduced pressure. The crude was re-dissolved in DCM (10 mL) and chromatographed on silica gel with hexane/EtOAc mixtures of increasing polarity (19.8:0.2 → 9.8:10.2). A mixture of **10a**/**10b** = 1.0/0.24 was obtained (1.97 g, 72% yield).

#### 3.2.2. Epoxidation of Alkene **9**. Obtaining (22*S*)-6-oxo-22,23-epoxy-24-nor-5α-cholan-3α-yl acetate and (**11a**) and (22*R*)-6-oxo-22,23-epoxy-24-nor-5α-cholan-3α-yl acetate (**11b**)

To a solution of alkene **9** (4.0 g, 10.36 mmol) in CH_2_Cl_2_ (100 mL) NaHCO_3_ (1.13 g, 13.45 mmol) and *m*CPBA (77%, 2.32 g, 13.45 mmol) were added. Then the mixture was stirred for 48 h and the end of the reaction was verified by TLC. Later, the reaction mixture was filtered to eliminate the *m*-chlorobenzoic acid formed and the liquid was evaporated under reduced pressure. The residue was dissolved in AcOEt (30 mL) and the solution was washed with a saturated solution of NaHCO_3_ (2 × 10 mL), then with water (3 × 10 mL), dried over MgSO_4_, and filtered. The solvent was evaporated under reduced pressure. The crude was re-dissolved in DCM (10 mL) and chromatographed on silica gel with hexane/EtOAc mixtures of increasing polarity (19.8:0.2 → 12.8:7.2). A mixture of **11a**/**11b** = 0.44/1.0 was obtained (2.32 g, 55.7% yield).

Compound **11a**: ^1^H-NMR (600 MHz, CDCl_3_) *δ* (ppm): 5.13–5.11 (1H, m, H-3), 2.64 (1H, dd, *J* = 5.1 and 4.1 Hz, H-23β), 2.63 (1H, ddd, *J* = 6.9, 4.1, and 2.8 Hz, H-22), 2.40 (1H, dd, *J* = 5.1 and 2.8 Hz, H-23α), 2.56 (1H, dd, *J* = 12.7 and 2.1 Hz, H-5), 2.33 (1H, dd, *J* = 13.2 and 4.6 Hz, H-7α), 2.03 (3H, s, CH_3_CO), 0.970 (3H, d, *J* = 6.8 Hz, H-21), 0.744 (3H, s, H-19), 0.667 (3H, s, H-18). ^13^C-NMR (150 MHz, CDCl_3_) see Table S1.

Compound **11b**: ^1^H-NMR (600 MHz, CDCl_3_) *δ* (ppm): 5.13–5.11(1H, m, H-3), 2.80 (1H, dd, *J* = 4.8 and 3.8 Hz, H-23β), 2.73 (1H, ddd, *J* = 6.5, 3.8, and 2.5 Hz, H-22), 2.59 (1H, dd, *J* = 4.8 and 2.7 Hz, H-23α), 2.56 (1H, dd, *J* = 12.7 and 2.1 Hz, H-5), 2.32 (1H, dd, *J* = 13.3 and 4.6 Hz, H-7α), 2.03 (3H, s, CH_3_CO), 1.11 (3H, d, *J* = 5.6 Hz, H-21), 0.744 (3H, s, H-19), 0.659 (3H, s, H-18). ^13^C-NMR (150 MHz, CDCl3) see Table S1.

#### 3.2.3. Opening of Epoxide Ring of Mixture **11a**/**11b** in Acid Medium. Obtaining (22*S*)-22,23-dihydroxy-6-oxo-24-nor-5α-cholan-3α-yl acetate (**10a**) and (22*R*)-22,23-dihydroxy-6-oxo-24-nor-5 α-cholan-3 α-yl acetate (**10b**)

To a solution of mixture **11a**/**11b** (2.28 g, 5.66 mmol) in THF (60 mL) and H_2_O (15 mL), *p*-TsOH (0.19 g, 1.10 mmol) was added. The mixture was stirred for 3 h and the end of the reaction was verified by TLC. Then the solvent was evaporated under reduced pressure and removed (15 mL approximate volume). The residue was dissolved in AcOEt (50 mL) and the solution was washed with a saturated solution of NaHCO_3_ (3 × 10 mL), then with water (3 × 10 mL), dried over MgSO_4_, and filtered. The solvent was evaporated under reduced pressure and the solid residue was crystallized from a 3:1 of Et_2_O/MeOH mixture. A mixture of **10a**/**10b** = 1.0:1.14 was obtained (1.80 g, 75.8% yield).

#### 3.2.4. Direct Asymmetric Sharpless Dihydroxylation of Alkene **9**. Obtaining (22*S*)-22,23-dihydroxy-6-oxo-24-nor-5α-cholan-3α-yl acetate (**10a**) and (22*R*)-22,23-dihydroxy-6-oxo-24-nor-5α-cholan-3α-yl acetate (**10b**)

To a mixture of *t*-butanol/water (40 mL, 1:1 *v/v*) alkene **9** (500 mg, 1.29 mmol), DHQD-CLB (157 mg, 0.331 mmol), CH_3_SO_2_NH_2_ (228 mg, 2.33 mmol), K_2_CO_3_ (993 mg, 7.11 mmol), and K_3_[Fe(CN)_6_] (2.494 g, 7.50 mmol) were added, then the mixture was homogenized by magnetic stirring for 5 min. Later, 700 μL of OsO_4_ solution (1.0 g, 3.933 mmol in 20 mL of *t*-butanol) were added, and the mixture reaction was stirred at room temperature for 6 h. The end of the reaction was verified by TLC, then H_2_O (10 mL) and a saturated solution of Na_2_S_2_O_3_·5H_2_O (10 mL) were added. The reaction mixture was extracted with AcOEt (2 × 30 mL), both organic phases were combined, dried over MgSO_4_, and filtered. The solvent was evaporated under reduced pressure. The crude was re-dissolved in DCM (10 mL) and chromatographed on silica gel with hexane/EtOAc mixtures of increasing polarity (19.8:0.2 → 9.8:10.2). A mixture of **10a**/**10b** = 1.0/0.9 was obtained (515 mg, 95% yield).

#### 3.2.5. Synthesis of (22*S*)-22-hydroxy-6-oxo-24-nor-5α-cholan-3α,23-diyl 3-acetate 23-benzoate (**5**), (22*R*)-22-hydroxy-6-oxo-24-nor-5α-cholan-3α,23-diyl 3-acetate 23-benzoate (**7**) and (22*S*)-6-oxo-24-nor-5α-cholan-3α,22,23-triyl 3-acetate 22,23-dibenzoate (**8**)

To a solution of mixture **10a**/**10b** (3.702 g, 8.216 mmol) in CH_2_Cl_2_ (75 mL), DMAP (20 mg, 0.819 mmol) and pyridine (5.0 mL, *d* = 0.981 g/mL) were added. The mixture was cooled to 0–5 °C under nitrogen atmosphere.

PhCOCl 1.10 mL (9.47 mmol, *d* = 1.21 g/mL) was added by slow drip during 1 h, then the reaction mixture was maintained at room temperature for 4 h. The end of the reaction was verified by TLC, the solvent was evaporated under reduced pressure until concentrated to a volume of 10 mL. Subsequently, the mixture was diluted with EtOAc (40 mL). The organic layer was washed with a saturated solution of KHSO_4_ (2 × 10 mL), H_2_O (3 × 30 mL), dried over MgSO_4_, and filtered. The solvent was evaporated under reduced pressure. The crude was re-dissolved in DCM (10 mL) and chromatographed on silica gel with hexane/EtOAc mixtures of increasing polarity (19.8:0.2 → 9.8:10.2), a mixture of 4.966 g corresponding to mono and dibenzoylated compounds was obtained. The chromatographic separation was repeated three times, and five fractions were obtained in the following order of polarity. Fraction I: compound **8** (283 mg, 5.7% yield); Fraction II: mixture of compounds **8** and **12** (3.960 g, **8**:**12** = 0.4:1.0, 79.9% yield); Fraction III: mixture of compounds **5** and **7** (248 mg, **5**:**7** = 0.7:1.0, 5.0% yield); Fraction IV: compound **7** (59 mg, 1.2% yield); Fraction V: compound **5** (198 mg, 4% yield).

Compound **5**: Physical properties (melting point) and spectroscopic data (IR, ^1^H, ^13^C-NMR) were consistent with those previously reported [35].

Compound **7**: colorless solid, m.p. 174.6 ± 1 °C (Hexane/Et_2_O), IR_ν__max_ (cm^−1^): 3509 (O–H), 2948–2869 (C–H), 1731 (C=O), 1718 (C=O), 1703 (C=O), 1603 (C=C Ar), 1580 (C=C Ar), 1451 and 1379 (C–H), 1277 (C–O), 1157 (C–O), 1088 (C–O), 712 (CH-Ar). ^1^H-NMR (400 MHz, CDCl_3_) *δ* (ppm): 8.02 (2H, d, *J* = 7.4 Hz, HAr-2′), 7.54 (1H, t, *J* = 7.4 Hz, HAr-4′), 7.41 (1H, t, *J* = 7.8 Hz, HAr-3′), 5.08 (1H, m, H-3), 4.36 (1H, dd, *J* = 11.2 and 8.4 Hz, H-23a), 4.23 (1H, dd, *J* = 11.2 and 3.6 Hz, H-23b), 4.04 (1H, dd, *J* = 8.4 and 3.4 Hz, H-22), 2.54 (1H, dd, *J* = 11.7 and 2.9 Hz, H-5), 2.28 (1H, dd, *J* = 13.4 and 4.6 Hz, H-7β), 2.01 (3H, s, CH_3_CO), 0.990 (3H, d, *J* = 6.1 Hz, H-21), 0.712 (3H, s, H-19), 0.668 (3H, s, H-18). ^13^C-NMR see Table S1. HRMS-ESI (positive mode): *m/z* calculated for C_32_H_44_O_6_: 524.3138 [M]^+^, found 525.3204 [M + 1]^+^.

Compound **8**: colorless solid, m.p. 205.9 ± 2 °C (Hexane/Et_2_O), IR_ν__max_ (cm^−1^): 2943–2867, (C–H), 1736 (C=O), 1717 (C=O), 1710 (C=O), 1602 (C=C Ar), 1380 (CH_3_), 1284 (C–O), 1112 (C–O), 1070 (C–O), 713 (CH-Ar). ^1^H-NMR (400 MHz, CDCl_3_) *δ* (ppm): 8.06 (2H, dd, *J* = 8.5 and 1.4 Hz, HAr-2″), 7.97 (2H, dd, *J* = 8.5 and 1.4 Hz, HAr-2′), 7.55–7.50 (2H, m, HAr-4″ and HAr-4′), 7.44 (2H, dd, *J* = 7.9 and 7.9 Hz, HAr-3″ and HAr-5″), 7.38 (2H, dd, *J* = 7.9 and 7.4 Hz, HAr-3′ and HAr-5′), 5.56 (1H, dt, *J* = 9.4 and 3.1 Hz, H-22), 5.12 (1H, bt, *J* = 2.6 Hz, H-3), 4.66 (1H, dd, *J* = 12.0 and 2.3 Hz, H-23a), 4.49 (1H, dd, *J* = 12.0 and 9.4 Hz, H-23b), 2.57 (1H, dd, *J* = 12.5 and 3.2 Hz, H-5), 2.34 (1H, dd, *J* = 13.0 and 4.6 Hz, H-7α); 2.05 (3H, s, CH_3_CO), 1.16 (3H, d, *J* = 7.0 Hz, H-21), 0.748 (3H, s, H-19), 0.710 (3H, s, H-18). ^13^C-NMR see Table S1. HRMS-ESI (positive mode): *m/z* calculated for C_39_H_48_O_7_: 628.3400 [M]^+^, found 629.3461 [M + 1]^+^.

#### 3.2.6. Synthesis of (22*S*)-24-nor-5α-cholan-6-oxo-3α,22,23-triyl 3-acetate 22,23-dibenzoate (**8**) from **5**

To obtain compound **8** from **5** the same procedure described above was followed, with **5** (100 mg, 0.191 mmol) in CH_2_Cl_2_ (20 mL), pyridine (1.0 mL), DMAP (0.5 mg), PhCOCl (0.5 mL, 4.40 mmol). Compound **8** (114 mg, 95% yield). The spectroscopic data of ^1^H and ^13^C-NMR were consistent with those described above.

#### 3.2.7. Synthesis of (22*S*)-3α,22,23-trihydroxy-24-nor-5α-cholan-6-one (**4**) from **5**

To a solution of **5** (50 mg, 2.46 mmol) in MeOH (30 mL) K_2_CO_3_ (10 mL, 15% *w/w*) was added. Then the suspension was stirred at reflux for 1.5 h. The end of the reaction was verified by TLC. Then the solvent was removed to dry and the residue acidified with 2.5% *w/w* HCl (5 mL). The obtained solid was filtered and washed with 5% NaHCO_3_ (10 mL) and water (2 × 10 mL) and dried. Compound **4** (32.5 mg, 90% yield). The physical properties (melting point) and spectroscopic IR, ^1^H, ^13^C-NMR were consistent with those previously reported [35,40].

#### 3.2.8. Synthesis of (22*R*)-3α,22,23-trihydroxy-24-nor-5α-cholan-6-one (**6**) from **7**

To obtain compound **6** from **7** the same procedure described above was followed, with **6** (87 mg, 0.17 mmol) in MeOH (30 mL), K_2_CO_3_ (15 mL, 15% *w/w*), reflux 1.5 h. Compound **6** (52 mg, 82.5% yield).

Compound **6**: colorless solid, m.p. 210.5 ± 1 °C (Et_2_O/MeOH), IR_νmax_ (cm^−1^): 3421 (O–H), 2941–2867, (C–H), 1703 (C=O), 1461 (C–H), 1380 (CH_3_), 1284 (C–O), 1051 (C–O). ^1^H-NMR (400 MHz, MeOD) *δ* (ppm): 4.04 (1H, bs, H-3), 3.73 (1H, bt, *J* = 6.5 Hz, H-22), 3.53 (1H, dd, *J* = 10.9 and 7.1 Hz, H-23a), 3.46 (1H, dd, *J* = 10.9 and 6.5 Hz, H-23b), 2.78 (1H, t, *J* = 7.9 Hz, H-5), 2.24 (1H, dd, *J* = 13.0 and 4.7 Hz, H-7α), 2.13 (1H, bt, *J* = 13.0 Hz, H-7β), 0.925 (3H, d, *J* = 6.3 Hz, H-21), 0.762 (3H, s, H-19), 0.753 (3H, s, H-18). ^13^C-NMR see Table S1. HRMS-ESI (negative mode): *m/z* calculated for: C_23_H_38_O_4_: 378.2770 [M]^+^; found 377.2702 [M − 1]^+^.

## 4. Conclusions

In this study, BRs analogs and benzoylated derivatives with *R* stereochemistry at C22 were synthesized. The first synthetic step involves the transformation of the terminal olefin **9** to vicinal diol function. This reaction was carried out by three different dihydroxylation methods, and the yields and mixture composition of glycol mixtures with configuration *R*/*S* in C22 were determined. The results indicate that all tested methods lead to mixtures of both epimeric glycols. The selectivity is highest for the Upjohn reaction, but the total yield of glycols mixture is largest for the Sharpless method, 95%. Thus, a selection of one of these methods should depend of the expected outcome for hydroxylation reaction. The benzoylation reaction of the mixture of glycols obtained by Sharpless dihydroxylation produced a complex mixture of mono- and dibenzoylated derivatives, where the known analogue **5** and the new analogs **7** and **8** were separated and purified. Additionally, the new BRs analog **6** was obtained by saponification of derivative **7**. All chemical structures of the compounds synthesized in this research were fully characterized by 1D and 2D NMR experiments. Finally, we hope that these new analogs of brassinosteroids with short lateral side chain of 24-nor-5α-cholane type, and oxygenated functions in the form of diols in C22*S*/*R* and C23 benzoates, will allow to elucidate if a configuration at C22 is a structural requirement for hormonal growth activity in plants.

## Figures and Tables

**Table 1:** Comparison between signals of ^1^H (400.1 MHz, CDCl_3_) and ^13^C (100.6 MHz, CDCl_3_) NMR for H/C21, H/C22, and H/C23a–b, for epimers **5** and **7**.

H/C Signal	Compound 5	Compound 7
H-21	1.06 ppm (3H, d, *J* = 6.9 Hz)	0.990 ppm (3H, d, *J* = 6.1 Hz)
H-22	4.06 ppm (1H, m)	4.04 ppm (1H, dd, *J* = 8.4 and 3.6 Hz)
H-23a	4.50 ppm (1H, dd, *J* = 11.4 and 1.7 Hz)	4.36 ppm (1H, dd, *J* = 11.2 and 8.4 Hz)
H-23b	4.20 ppm (1H, dd, *J* = 11.4 and 4.2 Hz)	4.23 ppm (1H, dd, *J* = 11.2 and 3.6 Hz)
C21	12.90 ppm	12.30 ppm
C22	71.77 ppm	71.40 ppm
C23	66.39 ppm	68.84 ppm

**Table 2:** Comparison between the signals of ^1^H (400.1 MHz, MeOD) and ^13^C (100.6 MHz, MeOD) NMR for H/C21, H/C22, and H/C23a–b, for the epimers glycol **4** and **6**.

H/C Signal	Triol 4	Triol 6
H-21	0.943 ppm (3H, d, *J* = 6.9 Hz)	0.925 ppm (3H, d, *J* = 6.3 Hz)
H-22	3.71 ppm (1H, dt, *J* = 8.9 and 3.2 Hz)	3.73 ppm (1H, ta, *J* = 6.5 Hz)
H-23a	3.60 ppm (1H, dd, *J* = 11.3 and 2.7 Hz)	3.53 ppm (1H, dd, *J* = 10.9 and 7.1 Hz)
H-23b	3.40 ppm (1H, dd, *J* = 11.3 and 8.9 Hz)	3.46 ppm (1H, dd, *J* = 10.9 and 6.5 Hz)
C21	13.42 ppm	12.37 ppm
C22	75.19 ppm	74.45 ppm
C23	63.19 ppm	65.53 ppm

## Supplementary Materials

The following are available online, **Figure S1**: NMR spectra of *(22R)*-3α,22,23-trihydroxy-24-nor-5α-cholan-6-one (**6**), **Figure S2**: NMR spectra (22*R*)-22-hydroxy-6-oxo-24-nor-5α-cholan-3α,23-diyl 3-acetate 23-benzoate (**7**), **Figure S3**: NMR spectra of (22*S*)-6-oxo-24-nor-5α-cholan-3α,22,23-triyl 3-acetate 22,23-dibenzoate *(**8**)*, **Figure S4**: ^1^H NMR spectrum of (22*S*)-22,23-dihydroxy-6-oxo-24-nor-5α-cholan-3α-yl acetate (**10a**) and (22*R*)-22,23-dihydroxy-6-oxo-24-nor-5α-cholan-3α-yl acetate *(***10b**) mixture obtained by Upjohn dihydroxylation, **Figure S5**: ^1^H NMR spectrum of (22*S*)-22,23-dihydroxy-6-oxo-24-nor-5α-cholan-3α-yl acetate (**10a**) and (22*R*)-22,23-dihydroxy-6-oxo-24-nor-5α-cholan-3α-yl acetate *(***10b***)* mixture obtained by opening of epoxide ring mixture **11a**/**11b**, **Figure S6**: ^1^H NMR spectrum of (22*S*)-22,23-dihydroxy-6-oxo-24-nor-5α-cholan-3α-yl acetate *(***10a**) and (22*R*)-22,23-dihydroxy-6-oxo-24-nor-5α-cholan-3α-yl acetate (**10b**) mixture obtained by Sharpless dihydroxylation, **Figure S7**: NMR spectra of (22*S*)-6-oxo-22,23-epoxy-24-nor-5α-cholan-3α-yl acetate and (**11a**) and (22*R*)-6-oxo-22,23-epoxy-24-nor-5α-cholan-3α-yl acetate (**11b**) mixture, **Figure S8**: ^1^H NMR spectrum of (22*S*)-22-hydroxy-6-oxo-24-nor-5α-cholan-3α,23-diyl 3-acetate 23-benzoate (**5**) and (22*R*)-22-hydroxy-6-oxo-24-nor-5α-cholan-3α,23-diyl 3-acetate 23-benzoate (**7**) mixture, **Figure S9**: ^1^H NMR spectrum of (22*S*)-6-oxo-24-nor-5α-cholan-3α,22,23-triyl 3-acetate 22,23-dibenzoate (**8**) and (22*R*)-6-oxo-24-nor-5α-cholan-3α,22,23-triyl 3-acetate 22,23-dibenzoate (**12**) mixture*,* **Figure S10**: HRMS-ESI of (22*R*)-3α,22,23-trihydroxy-24-nor-6-oxo-5α-cholan (**6**), **Figure S11**: HRMS-ESI of (22*R*)-22-hydroxy-24-nor-6-oxo-5α-cholan-3α,23-diyl 3-acetate 23-benzoate (**7**), **Figure S12**: HRMS-ESI of (22*S*)-6-oxo-24-nor-5α-cholan-3α,22,23-triyl 3-acetate 22,23-dibenzoate (**8**). **Table S1:** ^13^C NMR signals for compounds **4**–**8, 10a**–**10b, 11a** and **11b**.
