# Supplementary material for: Synthesis and Structural Determination of New Brassinosteroid 24-Nor-5α-Cholane Type Analogs
Source: Molecules. 2019 Dec 17;24(24):4612. doi: 10.3390/molecules24244612 (PMC6943489; doi:10.3390/molecules24244612)
Supplement: Supplementary file 1 [file molecules-24-04612-s001.pdf]

molecules

MDPI

Article

Synthesis and Structural Determination of New Brassinosteroid 24-nor-5α-cholane Type Analogs

Jocelyn Oyarce [1], Vanessa Aitken [1], César González [1], Karoll Ferrer [1], Andrés F. Olea [2], Teodor Parella [3] and Luis Espinoza Catalán [1,*]

[1] Departamento de Química, Universidad Técnica Federico Santa María, Avenida España 1680, Valparaíso 224000, Chile; jocelyn.oyarce@sansano.usm.cl (J.O.); vanessa.aitken.13@sansano.usm.cl (V.A.); Karoll.ferrer.14@sansano.usm.cl (K.F.); cesar.gonzalez@usm.cl (C.G.)

[2] Instituto de Ciencias Químicas Aplicadas, Facultad de Ingeniería, Universidad Autónoma de Chile, El Llano Subercaseaux 2801, Santiago 8900000, Chile; andres.olea@uautonoma.cl

[3] Teodor Parella, Servei de Ressonància Magnètica Nuclear, Universitat Autònoma de Barcelona, 08193 Bellaterra, Barcelona, Catalonia, Spain; teodor.parella@uab.cat

* Correspondence: luis.espinozac@usm.cl; Tel.: +56-32-2654425

The following are available online, **Figure S1**: NMR spectra of *(22R)*-3α,22,23-trihydroxy-24-nor-5α-cholan-6-one (**6**), **Figure S2**: NMR spectra (22*R*)-22-hydroxy-6-oxo-24-nor-5α-cholan-3α,23-diyl 3-acetate 23-benzoate (**7**), **Figure S3**: NMR spectra of (22*S*)-6-oxo-24-nor-5α-cholan-3α,22,23-triyl 3-acetate 22,23-dibenzoate *(8)*, **Figure S4**: ^{1}H NMR spectrum of (22*S*)-22,23-dihydroxy-6-oxo-24-nor-5α-cholan-3α-yl acetate (**10a**) and (22*R*)-22,23-dihydroxy-6-oxo-24-nor-5α-cholan-3α-yl acetate *(***10b**) mixture obtained by Upjohn dihydroxylation, **Figure S5**: ^{1}H NMR spectrum of (22*S*)-22,23-dihydroxy-6-oxo-24-nor-5α-cholan-3α-yl acetate (**10a**) and (22*R*)-22,23-dihydroxy-6-oxo-24-nor-5α-cholan-3α-yl acetate *(***10b***)* mixture obtained by opening of epoxide ring mixture **11a/11b**, **Figure S6**: ^{1}H NMR spectrum of (22*S*)-22,23-dihydroxy-6-oxo-24-nor-5α-cholan-3α-yl acetate *(***10a**) and (22*R*)-22,23-dihydroxy-6-oxo-24-nor-5α-cholan-3α-yl acetate (**10b**) mixture obtained by Sharpless dihydroxylation, **Figure S7**: NMR spectra of (22*S*)-6-oxo-22,23-epoxy-24-nor-5α-cholan-3α-yl acetate and (**11a**) and (22*R*)-6-oxo-22,23-epoxy-24-nor-5α-cholan-3α-yl acetate (**11b**) mixture, **Figure S8**: ^{1}H NMR spectrum of (22*S*)-22-hydroxy-6-oxo-24-nor-5α-cholan-3α,23-diyl 3-acetate 23-benzoate (**5**) and (22*R*)-22-hydroxy-6-oxo-24-nor-5α-cholan-3α,23-diyl 3-acetate 23-benzoate (**7**) mixture, **Figure S9**: ^{1}H NMR spectrum of (22*S*)-6-oxo-24-nor-5α-cholan-3α,22,23-triyl 3-acetate 22,23-dibenzoate (**8**) and (22*R*)-6-oxo-24-nor-5α-cholan-3α,22,23-triyl 3-acetate 22,23-dibenzoate (**12**) mixture, **Figure S10**: HRMS-ESI of (22*R*)-3α,22,23-trihydroxy-24-nor-6-oxo-5α-cholan (**6**). **Figure S11**: HRMS-ESI of (22*R*)-22-hydroxy-24-nor-6-oxo-5α-cholan-3α,23-diyl 3-acetate 23-benzoate (**7**), **Figure S12**: HRMS-ESI of (22*S*)-6-oxo-24-nor-5α-cholan-3α,22,23-triyl 3-acetate 22,23-dibenzoate (**8**).**Table S1:** ^{13}C NMR signals for compounds **4–8, 10a–10b, 11a** and **11b**.

Figure S1. NMR spectra of (22*R*)-3α,22,23-trihydroxy-24-nor-5α-cholan-6-one (**6**).

^{1}H-NMR

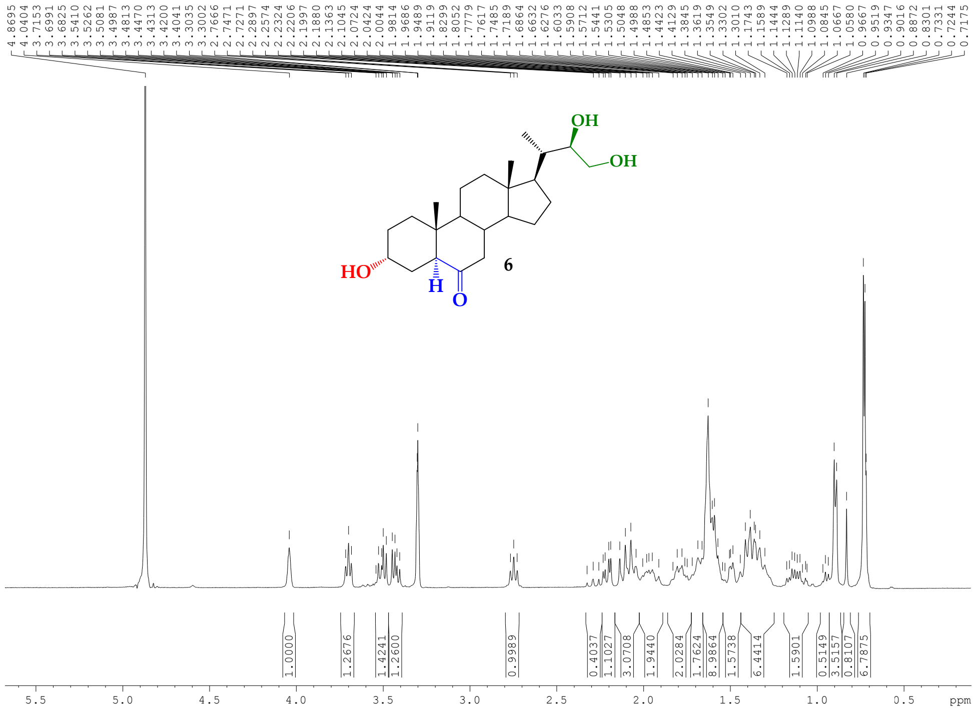

^{13}C-NMR

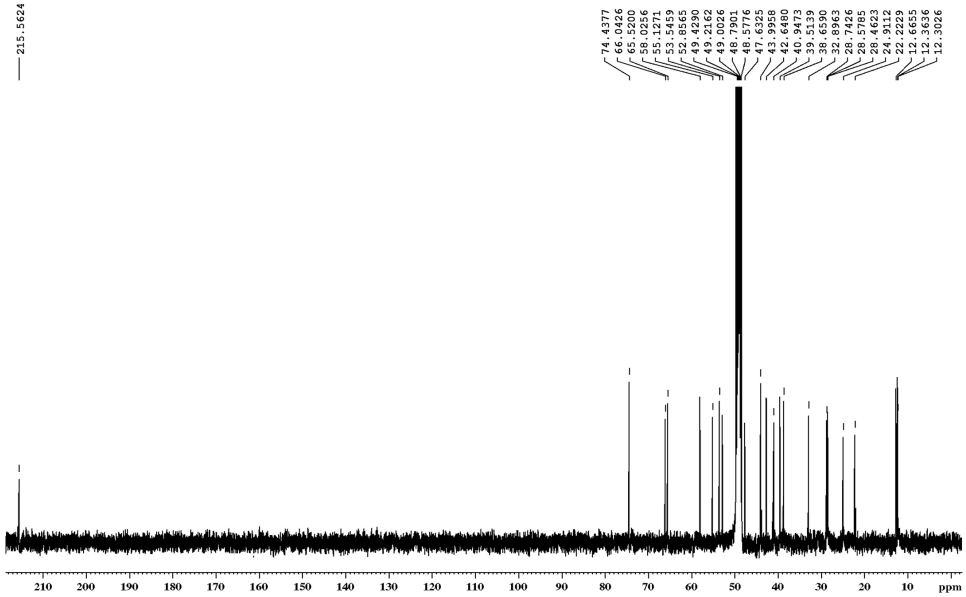

^{13}C DEPT-135 NMR

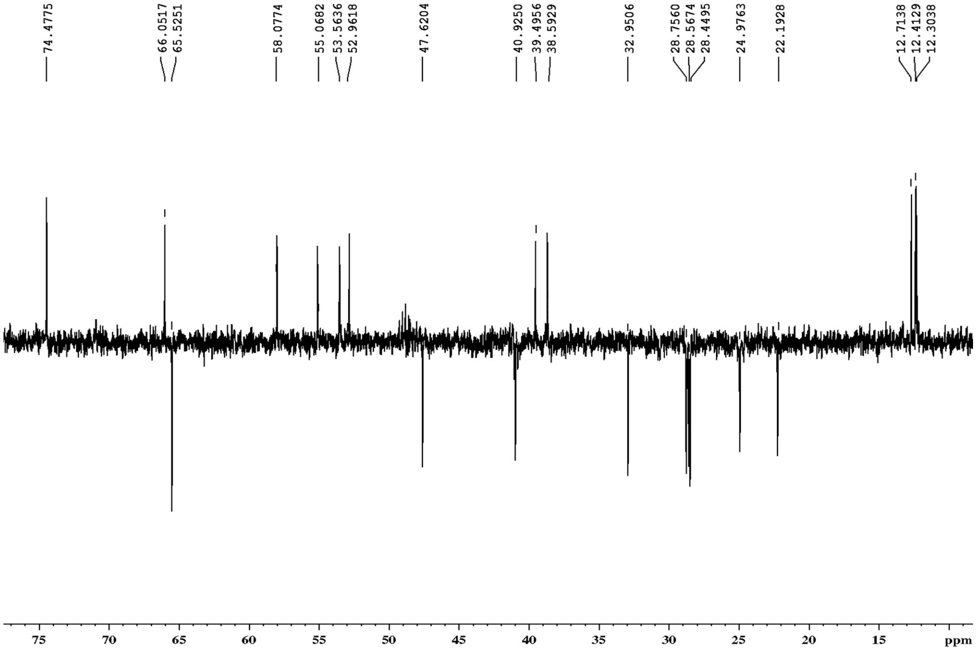

^{1}H-^{13}C 2D HSQC NMR

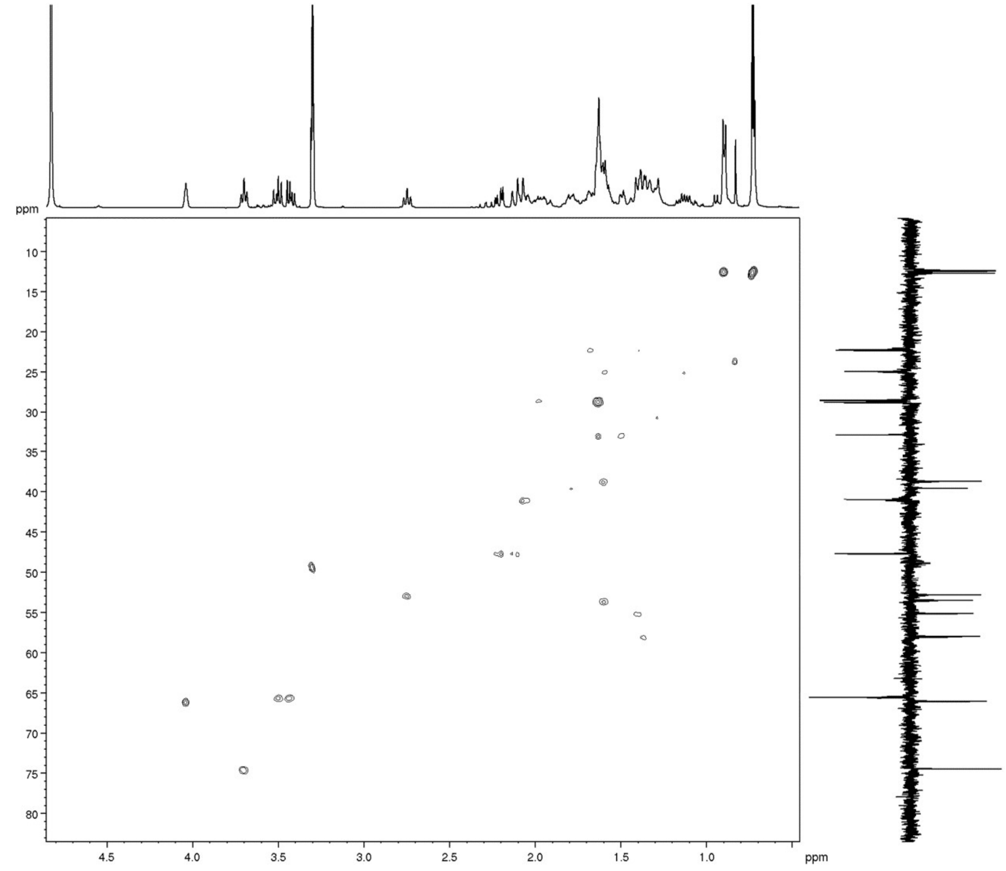

^{1}H-^{13}C 2D HMBC NMR

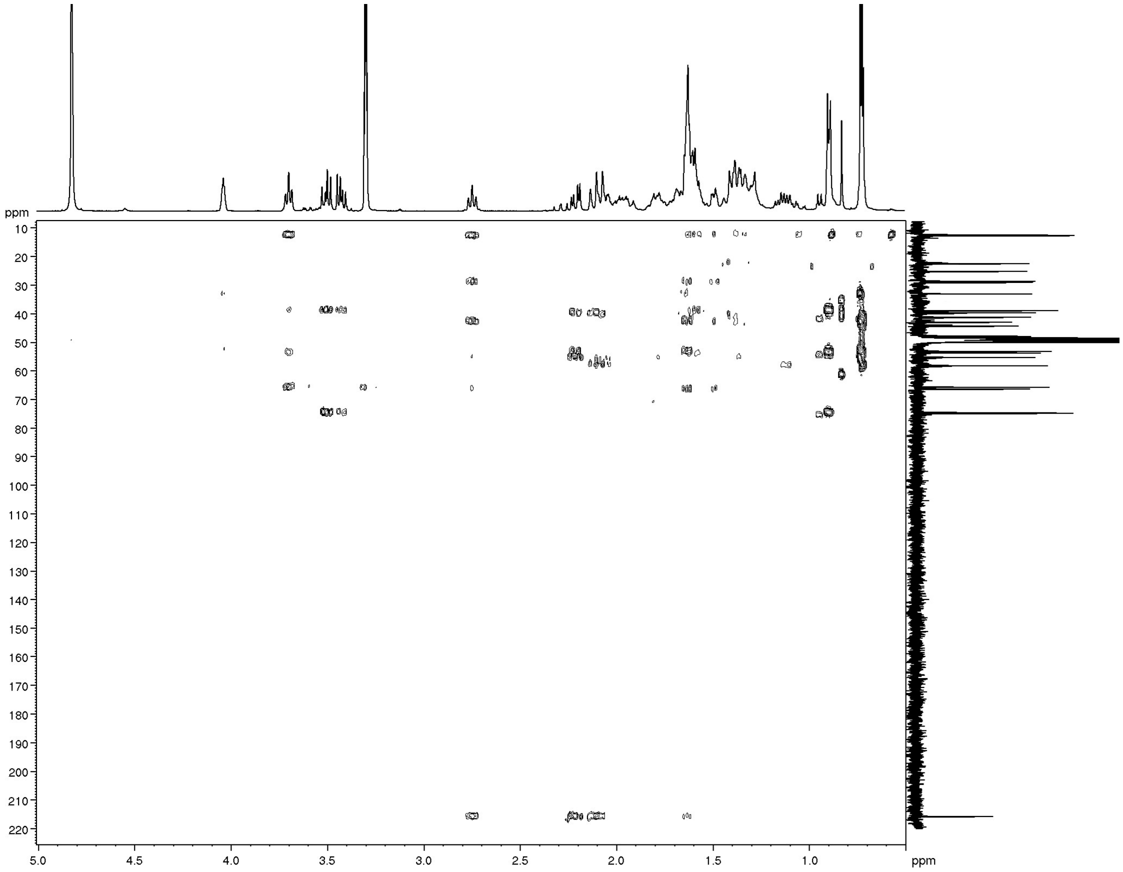

Figure S2. NMR spectra of spectra (22*R*)-22-hydroxy-6-oxo-24-nor-5α-cholan-3α,23-diyl 3-acetate 23-benzoate (**7**).

¹H-NMR

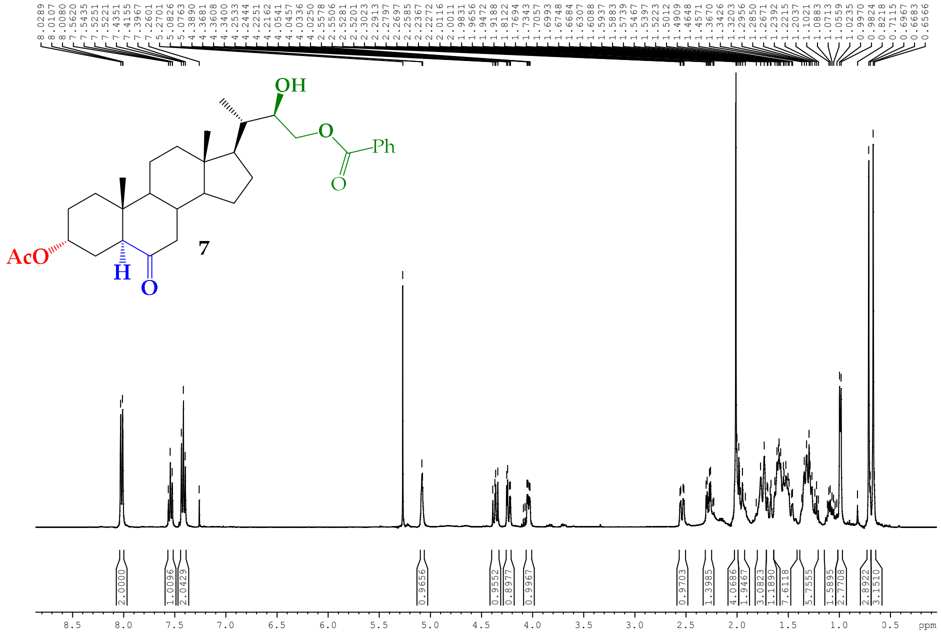

^{13}C-NMR

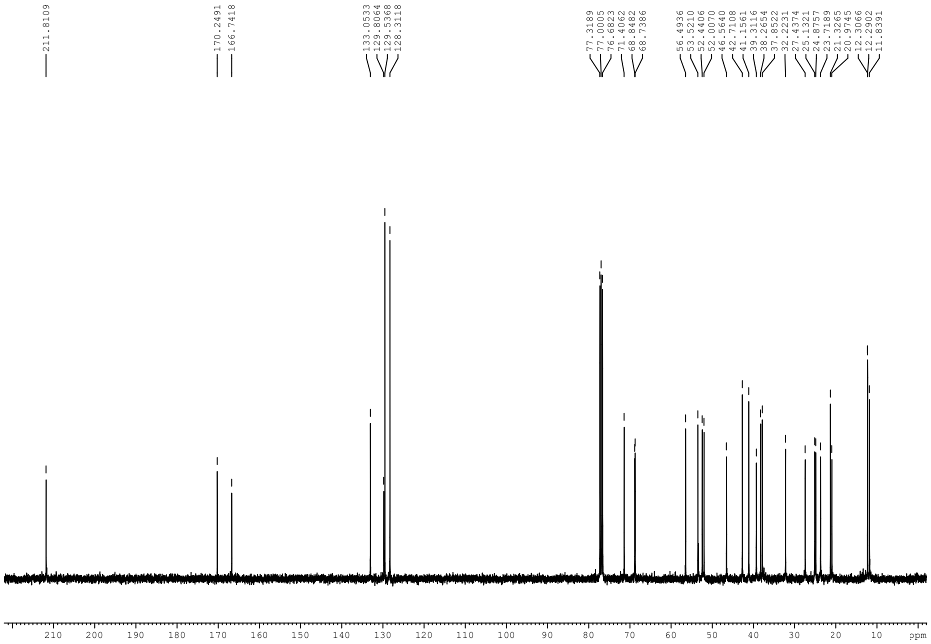

^{13}C DEPT-135 NMR

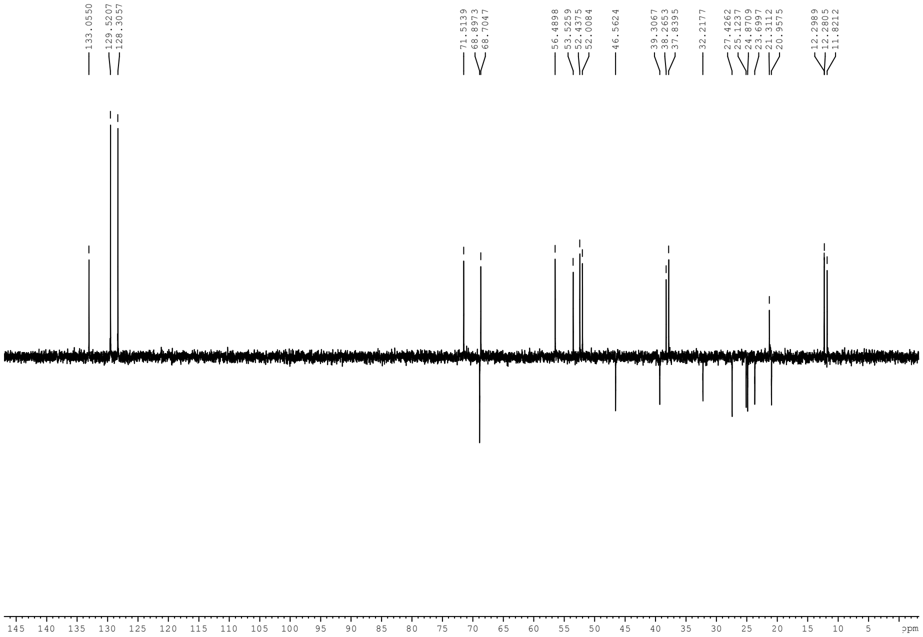

^{1}H-^{13}C 2D HSQC NMR

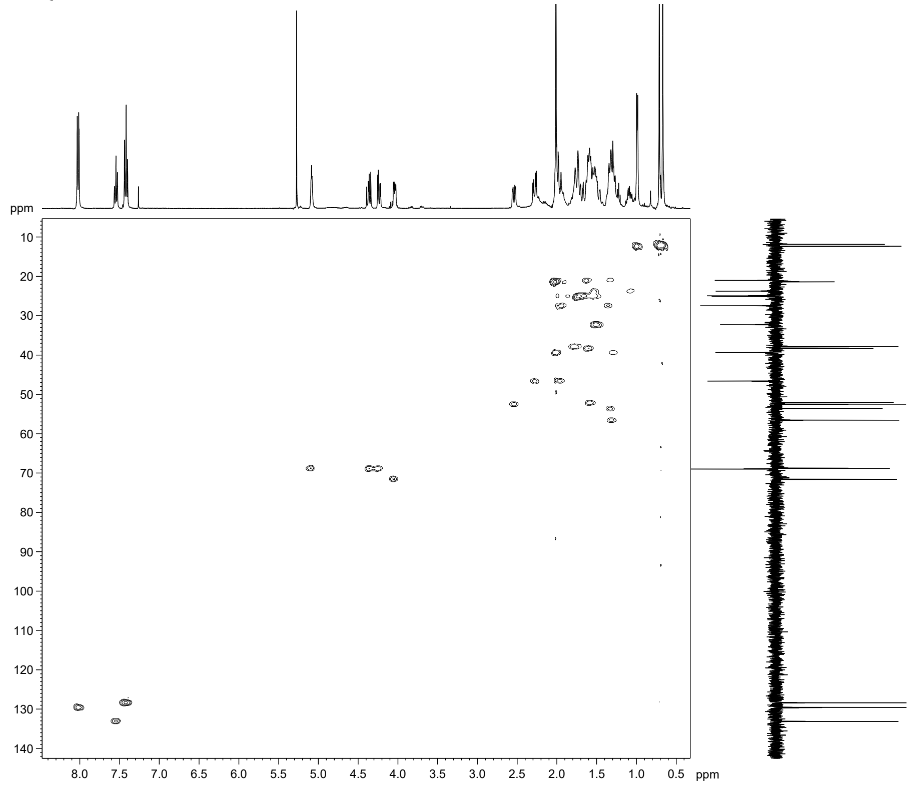

^{1}H-^{13}C 2D HMBC NMR

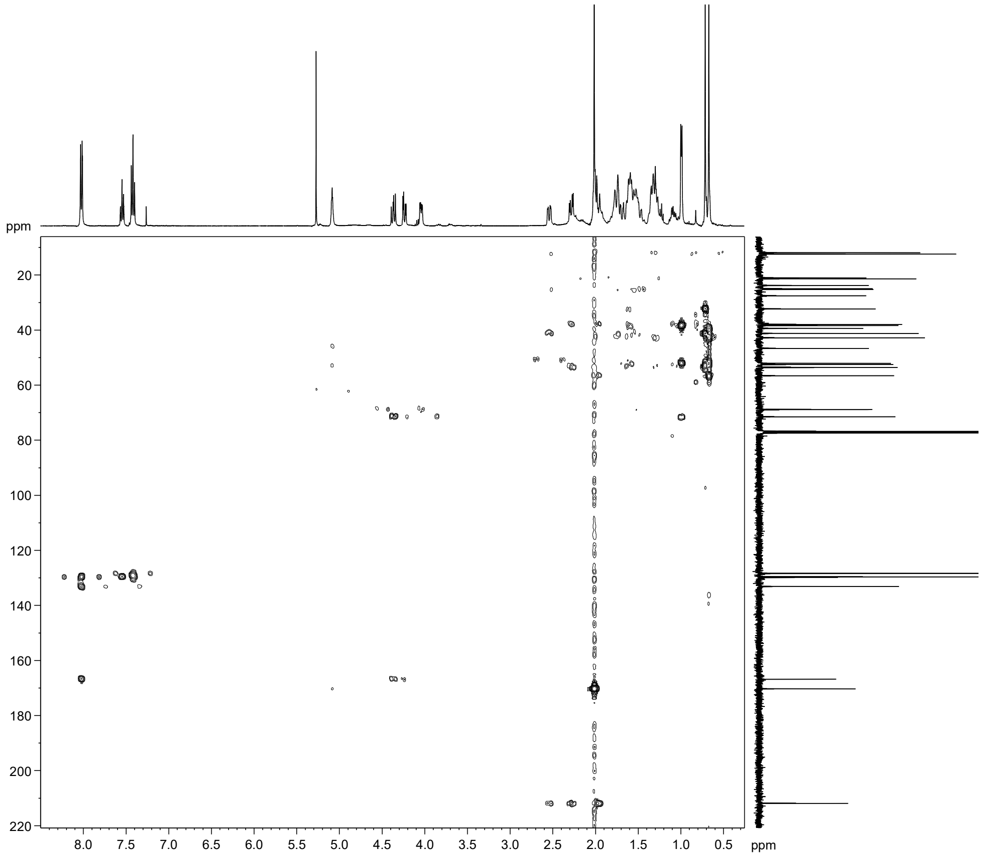

Figure S3. NMR spectra of (22*S*)-6-oxo-24-nor-5α-cholan-3α,22,23-triyl 3-acetate 22,23-dibenzoate (**8**).

^{1}H-NMR

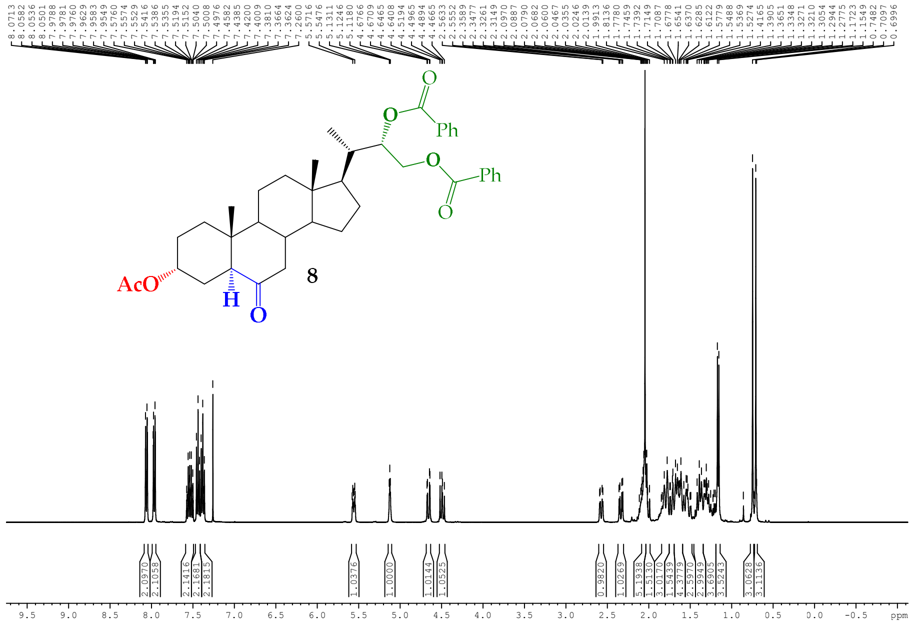

^{13}C-NMR

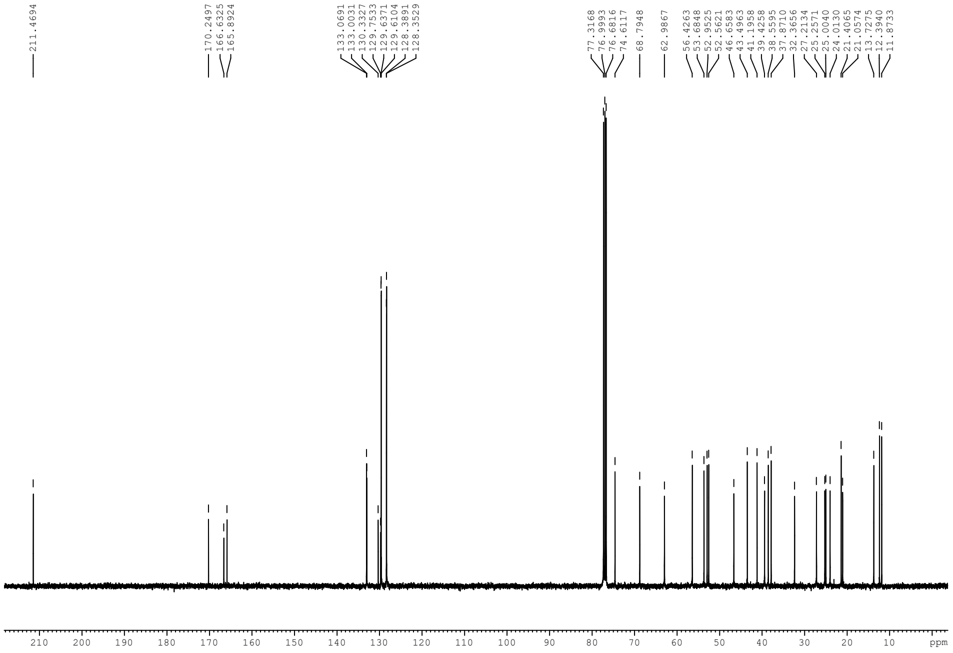

^{13}C DEPT-135 NMR

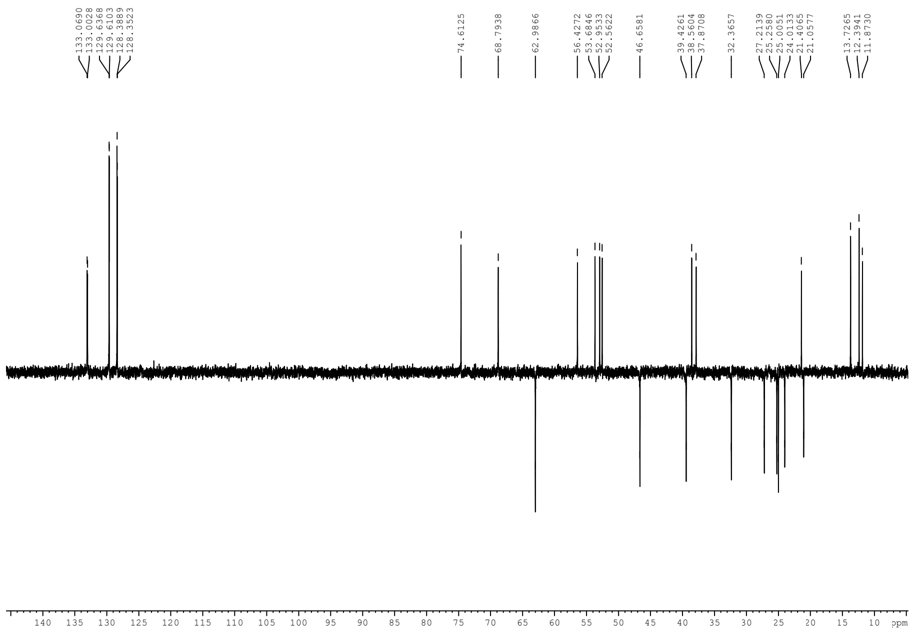

^{1}H-^{13}C 2D HSQC NMR

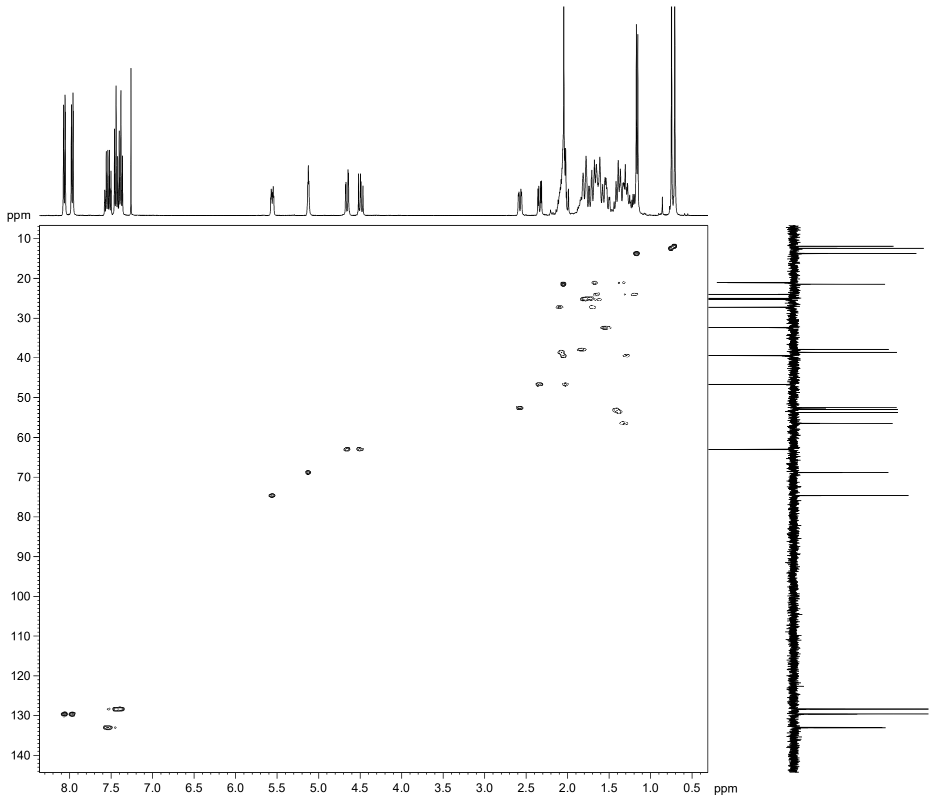

^{1}H-^{13}C 2D HMBC NMR

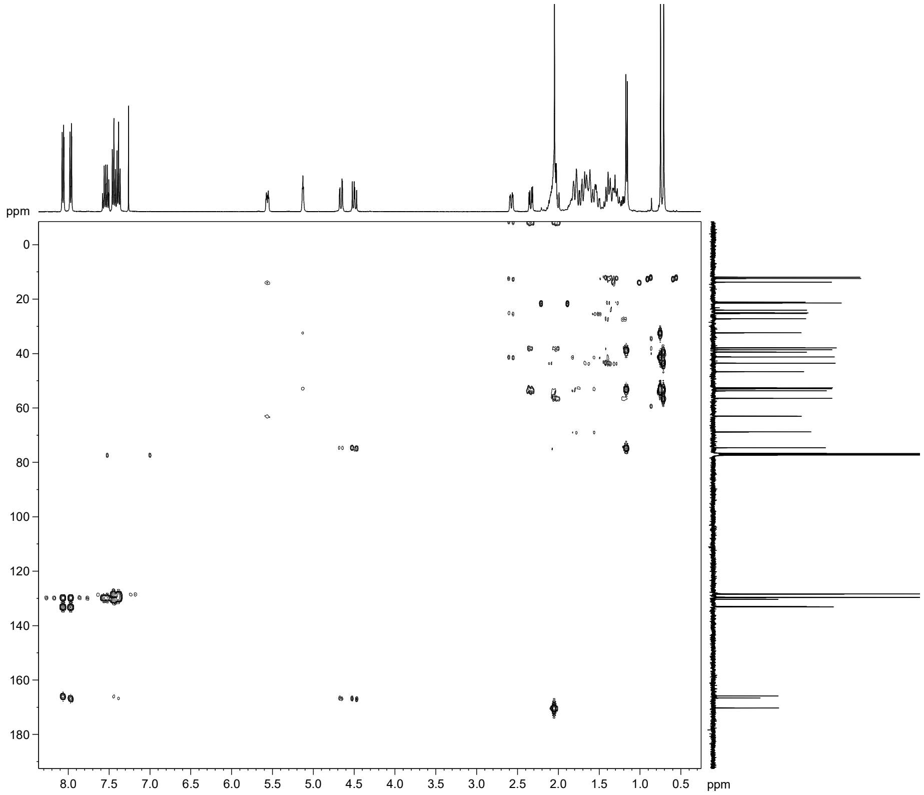

Figure S4. ^{1}H NMR spectrum of (22*S*)-22,23-dihydroxy-6-oxo-24-nor-5α-cholan-3α-yl acetate (**10a**) and (22*R*)-22,23-dihydroxy-6-oxo-24-nor-5α-cholan-3α-yl acetate (**10b**) mixture obtained by Upjohn dihydroxylation.

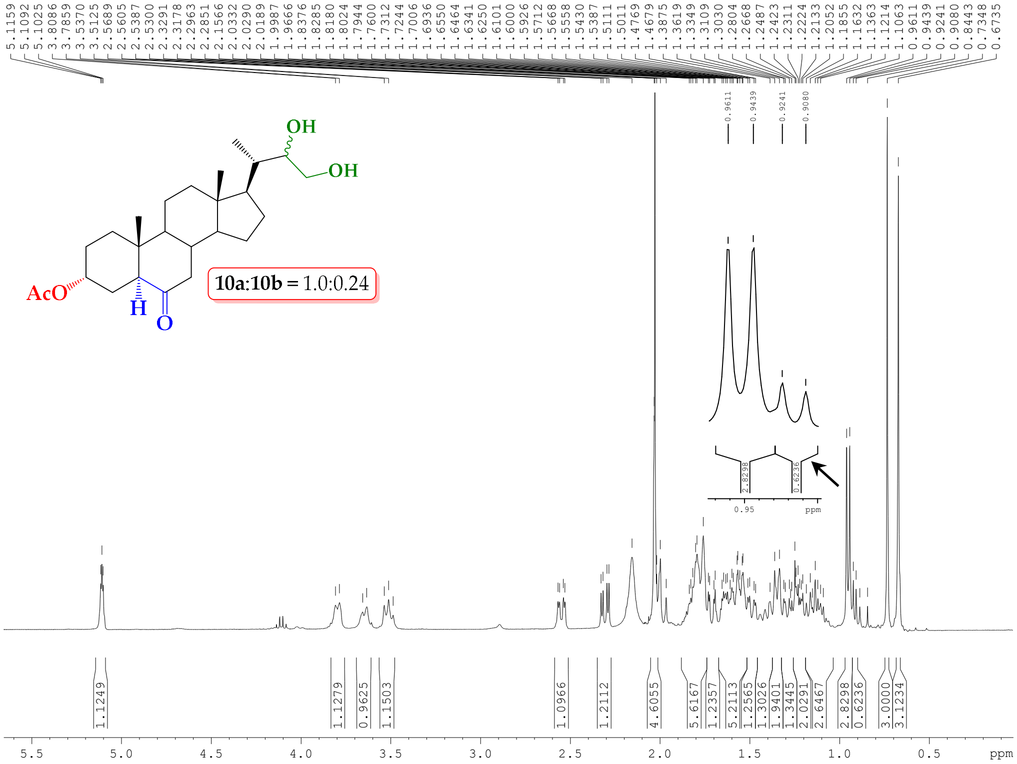

Figure S5. ^{1}H NMR spectrum of (22*S*)-22,23-dihydroxy-6-oxo-24-nor-5α-cholan-3α-yl acetate (**10a**) and (22*R*)-22,23-dihydroxy-6-oxo-24-nor-5α-cholan-3α-yl acetate (**10b**) mixture obtained by opening of epoxide ring mixture **11a**/**11b**.

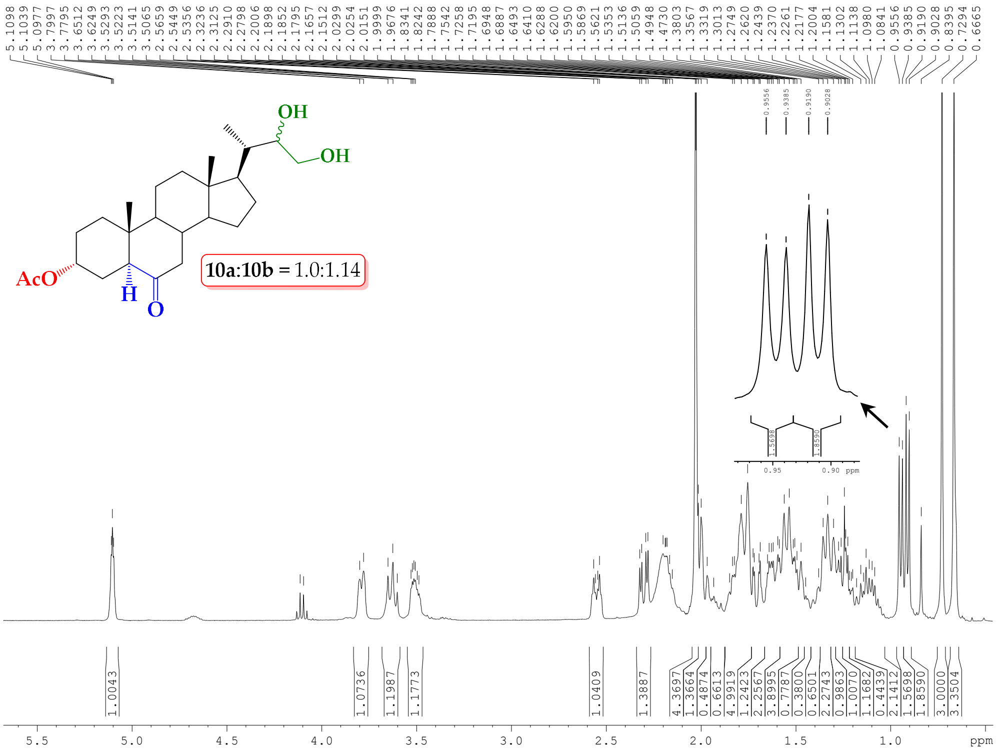

Figure S6. ^{1}H NMR spectrum of (22*S*)-22,23-dihydroxy-6-oxo-24-nor-5α-cholan-3α-yl acetate (**10a**) and (22*R*)-22,23-dihydroxy-6-oxo-24-nor-5α-cholan-3α-yl acetate (**10b**) mixture obtained by Sharpless dihydroxylation.

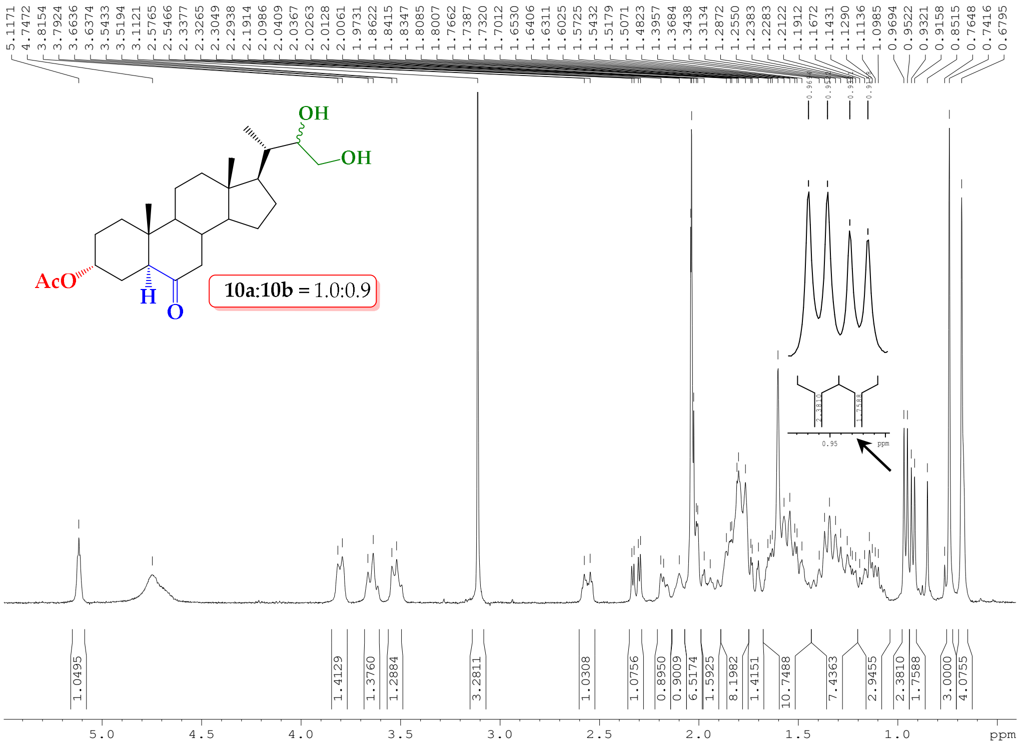

Figure S7. NMR spectra of (22*S*)-6-oxo-22,23-epoxy-24-nor-5α-cholan-3α-yl acetate and (**11a**) (22*R*)-6-oxo-22,23-epoxy-24-nor-5α-cholan-3α-yl acetate (**11b**) mixture.

^{1}H-NMR

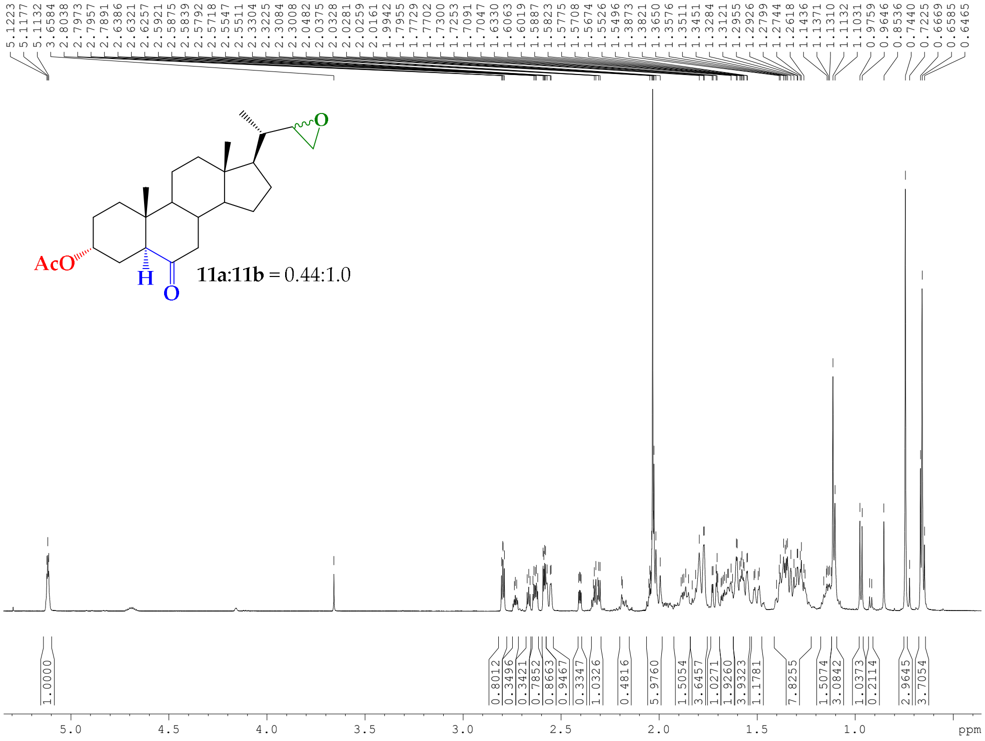

^{1}H NMR expansion 2,98-2,23 ppm

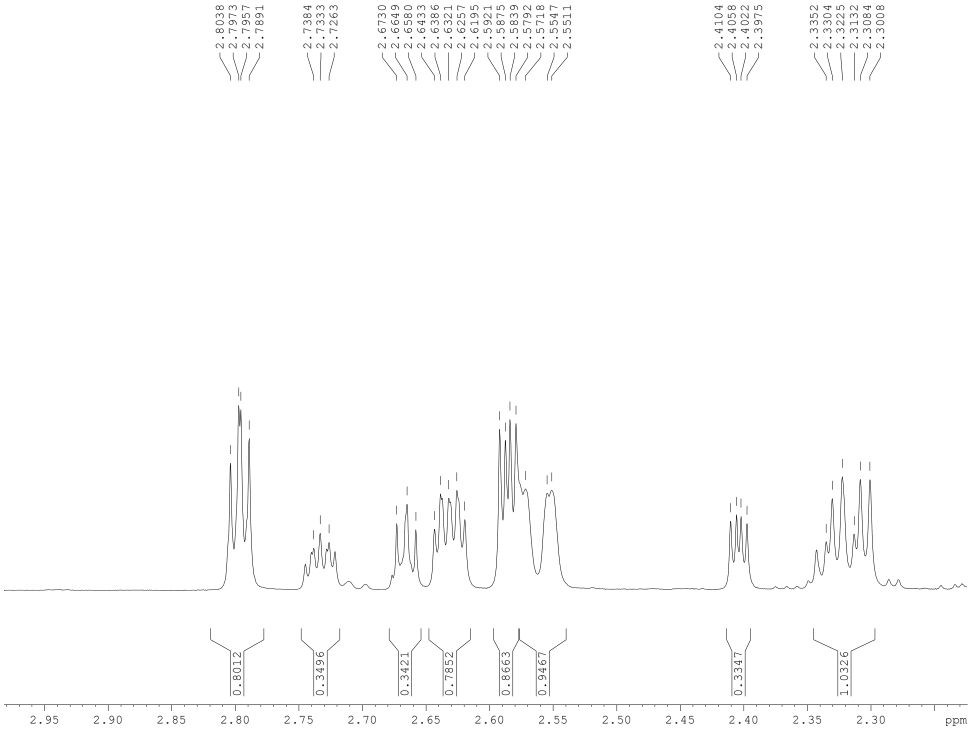

^{13}C-NMR

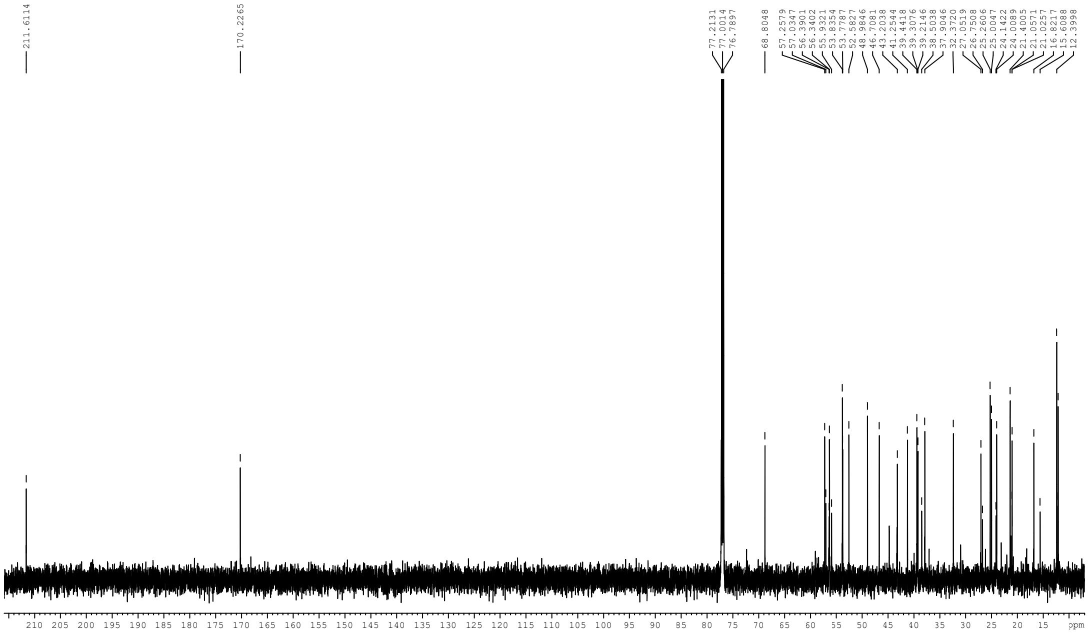

^{13}C NMR expansion 71-11 ppm

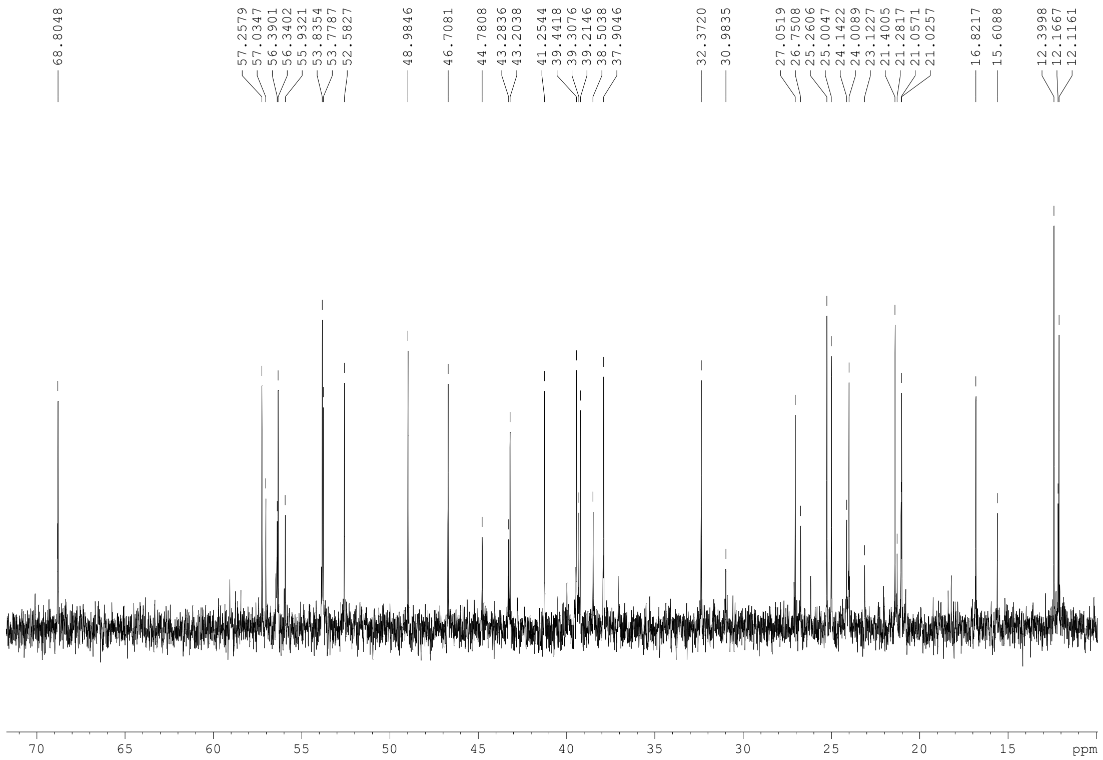

^{1}H-^{13}C 2D Edit HSQC NMR

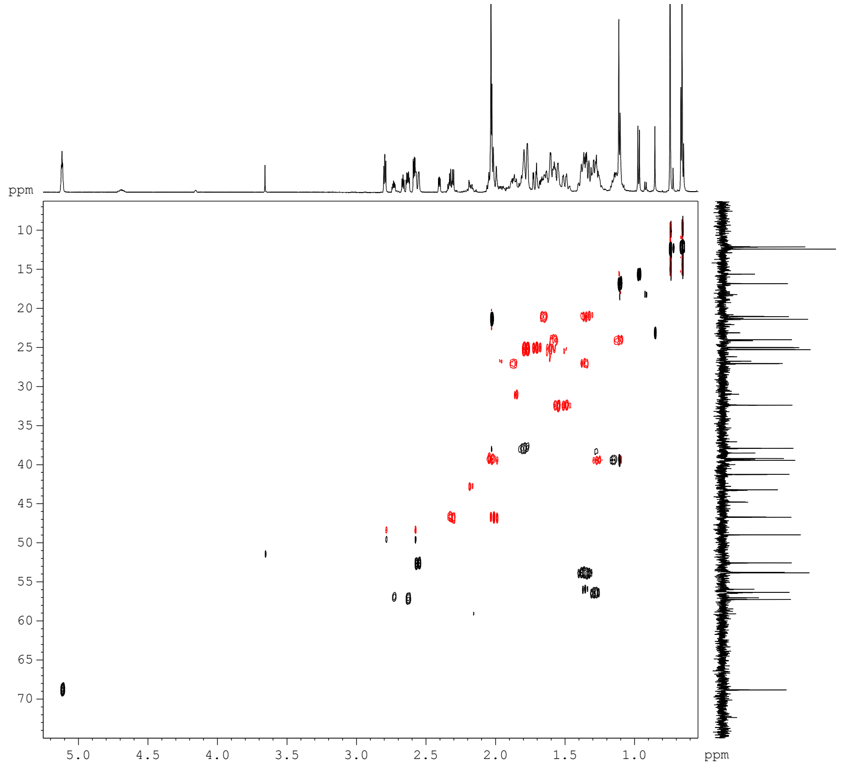

^{1}H-^{13}C 2D HMBC NMR

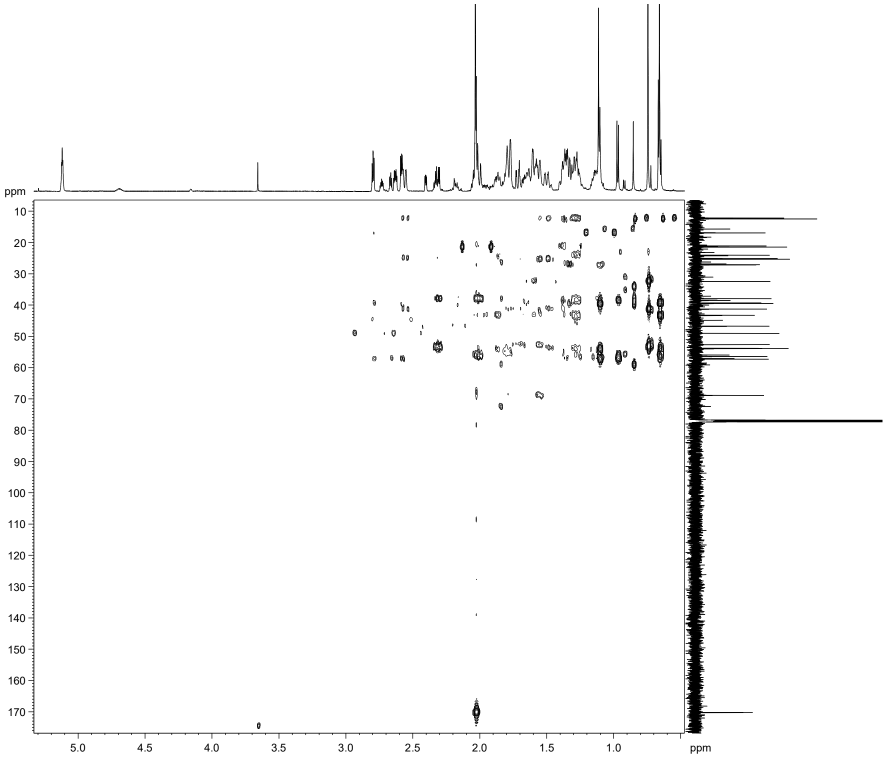

Figure S8. ^{1}H NMR spectrum of (22*S*)-22-hydroxy-6-oxo-24-nor-5α-cholan-3α,23-diyl 3-acetate 23-benzoate (**5**) and (22*R*)-22-hydroxy-6-oxo-24-nor-5α-cholan-3α,23-diyl 3-acetate 23-benzoate (**7**) mixture.

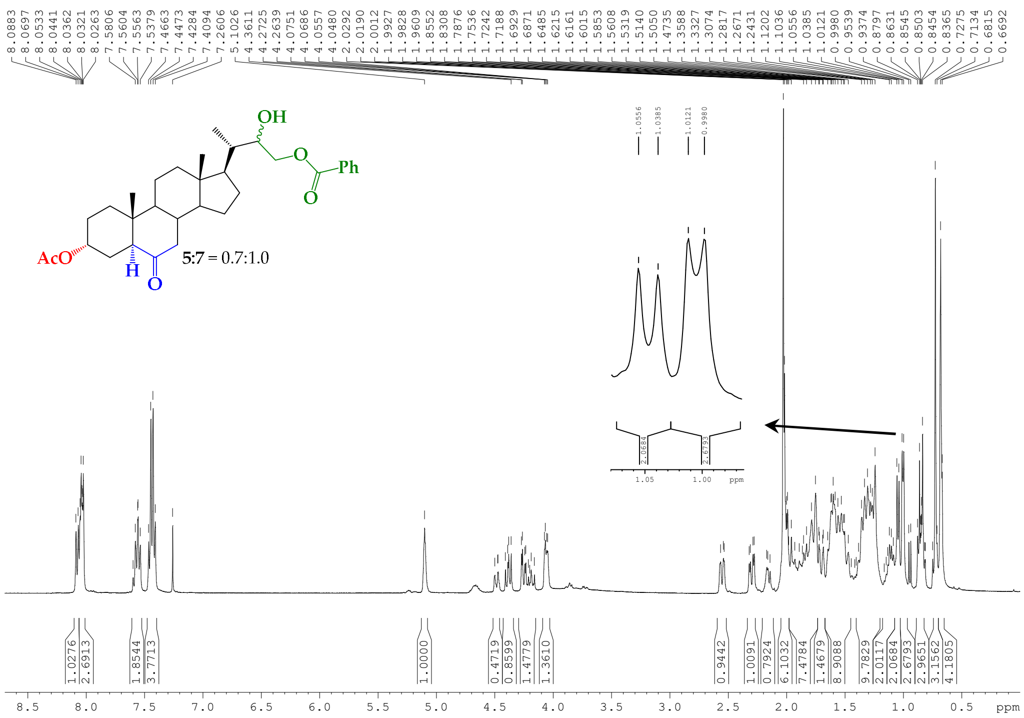

Figure S9. ¹H NMR spectrum of (22*S*)-24-nor-5α-cholan-6-oxo-3α,22,23-triyl 3-acetate 22,23-dibenzoate (**8**) and (22*R*)-24-nor-5α-cholan-6-oxo-3α,22,23-triyl 3-acetate 22,23-dibenzoate (**12**) mixture.

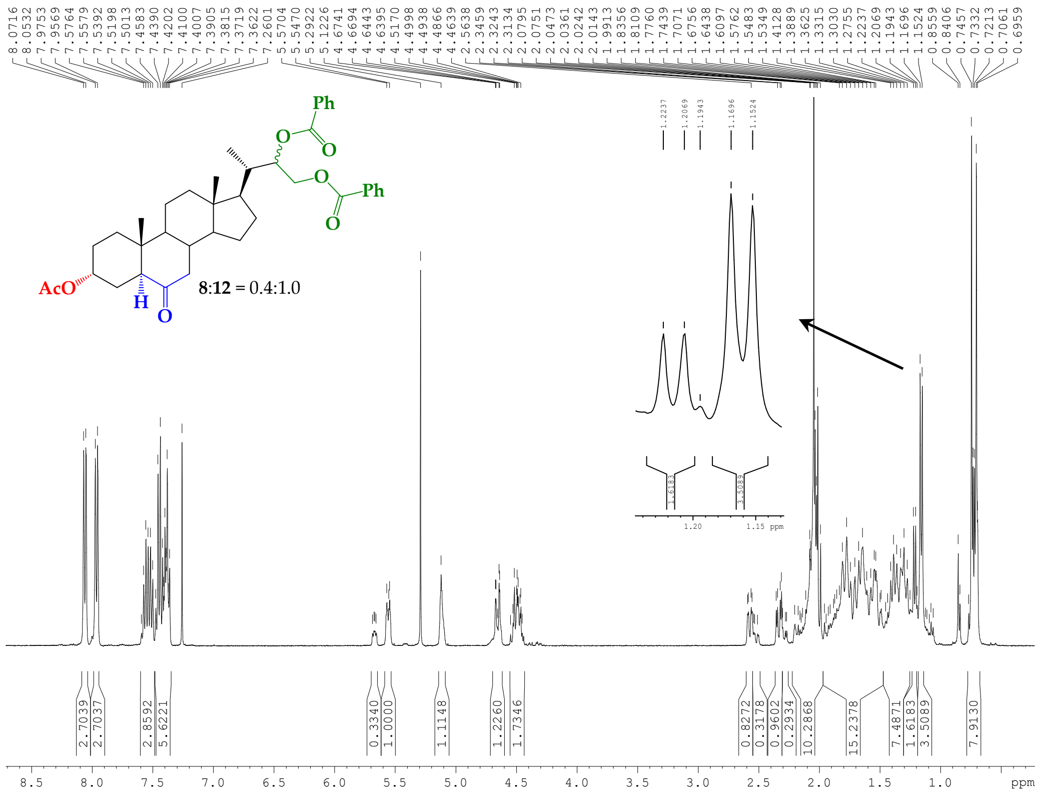

Figure S10. HRMS-ESI of (22*R*)-3α,22,23-trihydroxy-24-nor-5α-cholan-6-one (6).

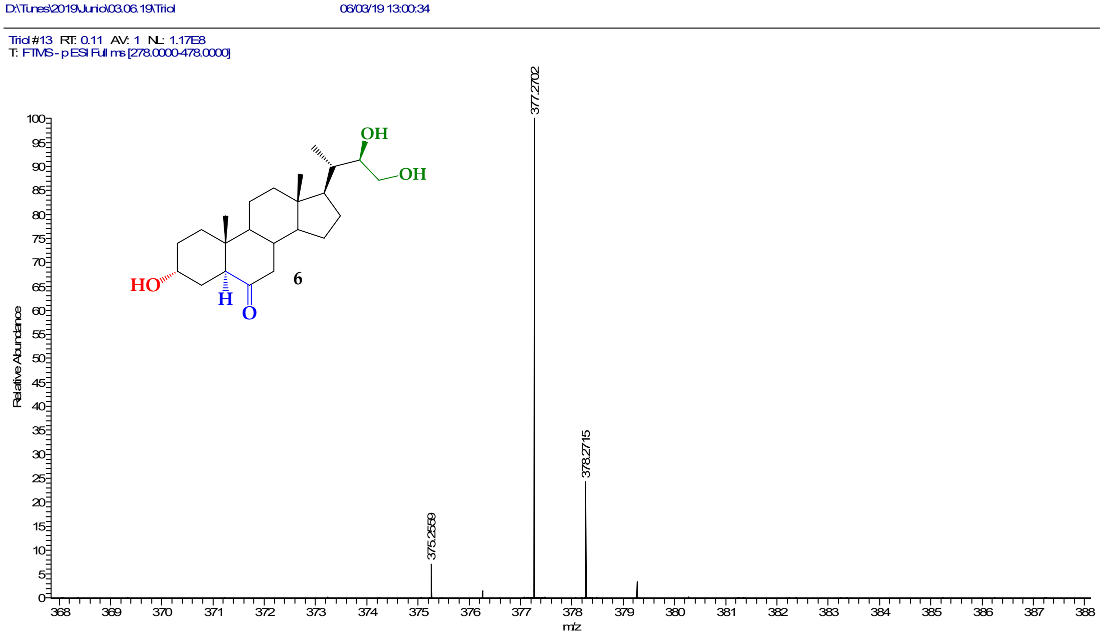

Figure S11. HRMS-ESI of (22*R*)-22-hydroxy-6-oxo-24-nor-5α-cholan-3α,23-diyl 3-acetate 23-benzoate (**7**).

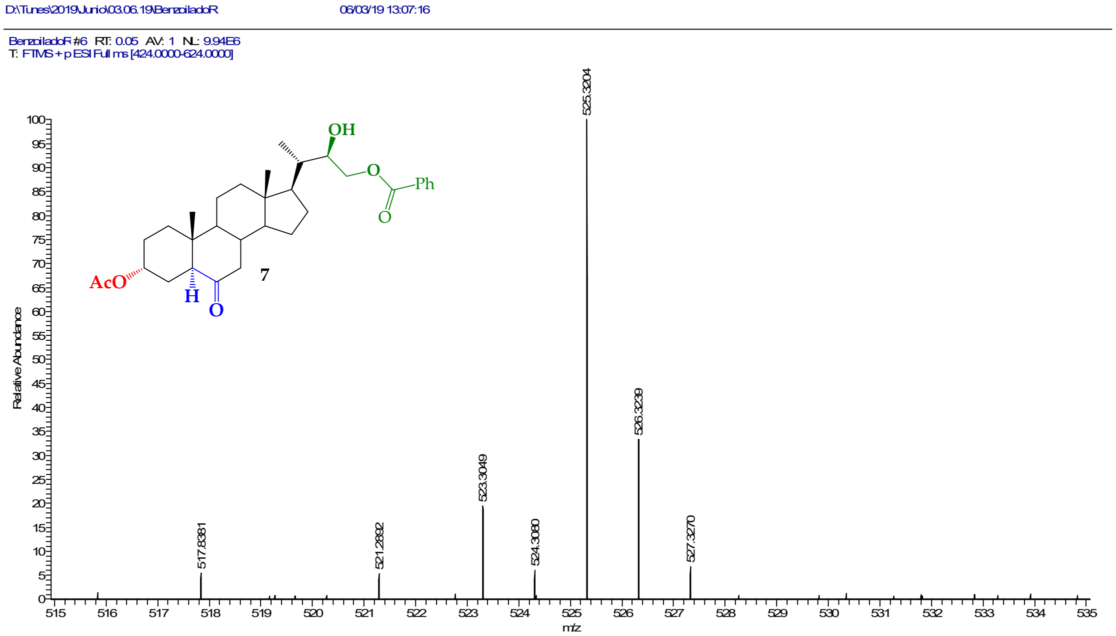

Figure S12. HRMS-ESI of (22*S*)-6-oxo-24-nor-5α-cholan-3α,22,23-triyl 3-acetate 22,23-dibenzoate (**8**).

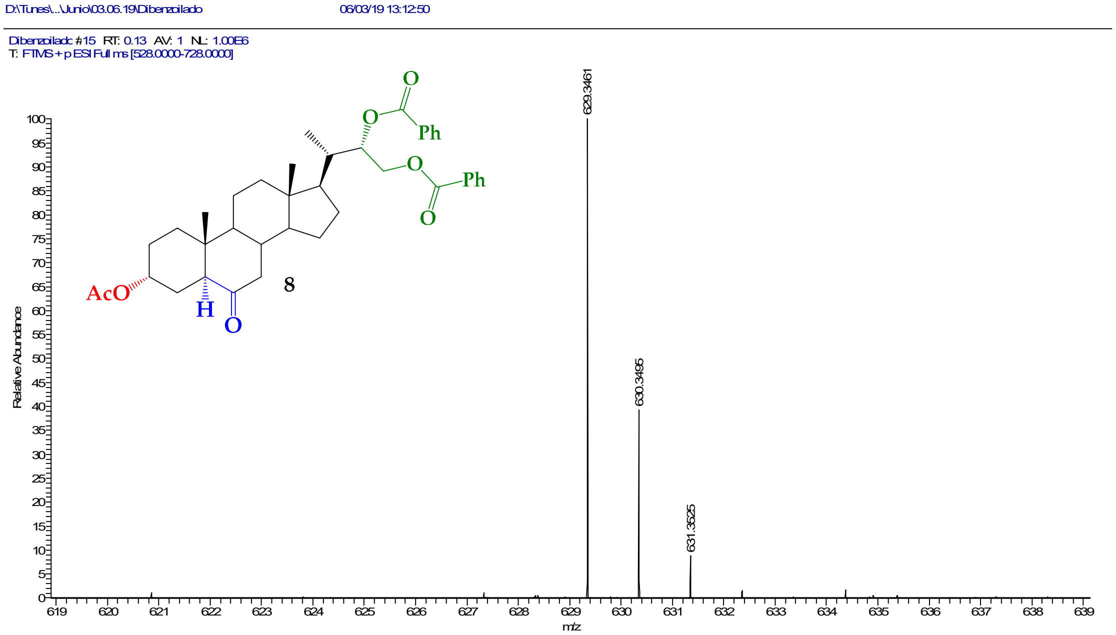

Table S1. ^{13}C NMR signals for compounds 4-8, 10a-10b, 11a and 11b.

Table S1. δ (ppm) ^{13}C NMR for compounds **4-8**, **10a-10b**, **11a** and **11b**.

N° C	4*	5†	6*	7†	8†	10a†	10b†	11a°	11b°
1	32.88	32.36	32.90	32.22	32.36	32.17	32.34	32.45	32.45
2	28.73	27.41	28.75	27.43	27.21	27.20	27.64	27.06	27.06
3	66.02	68.81	66.04	68.73	68.79	68.70	68.81	68.83	68.83
4	28.53	25.26	28.58	25.13	25.25	25.05	25.22	25.26	25.26
5	52.84	52.58	52.86	52.44	52.55	52.38	52.56	52.59	52.59
6	214.49	211.62	215.55	211.81	211.46	211.76	211.72	211.63	211.61
7	47.61	46.69	47.63	46.56	46.65	46.49	46.66	46.72	46.71
8	39.45	37.90	39.52	37.85	37.86	37.74	37.88	37.93	37.91
9	54.19	52.90	53.56	52.00	52.94	52.68	52.56	53.78	53.83
10	42.62	41.23	42.64	41.15	41.49	41.07	41.21	41.26	41.26
11	22.19	21.06	22.22	20.97	21.06	20.87	21.03	21.06	21.03
12	40.76	39.41	40.94	39.31	39.41	39.19	39.37	39.31	39.44
13	44.46	43.41	44.00	42.71	43.49	43.16	43.45	41.26	41.26
14	55.08	53.72	55.13	53.52	53.68	53.51	53.71	55.99	55.94
15	28.45	25.00	28.47	24.87	25.00	24.78	24.96	25.00	25.00
16	25.07	24.02	24.91	23.71	24,00	23.85	23.87	24.14	24.01
17	57.56	56.41	58.03	56.49	56.41	56.15	56.32	56.39	56.39
18	12.14	11.81	12.30	11.83	11.87	11.56	11.71	12.17	12.12
19	12.67	12.40	12.67	12.29	12.39	12.20	13.36	12.40	12.40
20	42.01	40.33	38.66	38.26	38.55	39.87	39.90	39.31	39.22
21	13.42	12.90	12.37	12.30	13.72	12.86	20.80	15.61	16.82
22	75.19	71.77	74.45	71.40	74.60	73.69	73.99	57.03	57.26
23	63.19	66.39	65.53	68.84	62.98	62.22	62.36	44.78	48.99
$\underline{C}H_3CO$		21.41		21.32	21.40	21.21	21.24	21.40	21.40
$CH_3\underline{C}O$		170.27		170.24	170.24	170.15	170.15	170.23	170.23
C22-$\underline{C}$O-Ar					166.62				
C23-$\underline{C}$O-Ar		167.01		166.74	165.88				
C1′-Ar		129.86		129.80	130.33				
C4′-Ar		133.22		133.05	133.06				
C2′-Ar and C6′-Ar		129.63		129.53	129.60				
C3′-Ar and C5′-Ar		128.45		128.31	128.35				
C1′′-Ar					129.74				
C4′′-Ar					133.00				
*C2′′-Ar and C6′′-Ar					129.63				
*C3′′-Ar and C5′′-Ar					128.38				

* MeOD, 100.6 MHz; † $CDCl_3$, 100.6 MHz; ° $CDCl_3$, 150.9 MHz.
